# Socioeconomic inequalities in type 2 diabetes comorbidities in different population subgroups: trend analyses using German health insurance data

**DOI:** 10.1038/s41598-023-37951-y

**Published:** 2023-07-05

**Authors:** Batoul Safieddine, Stefanie Sperlich, Johannes Beller, Karin Lange, Siegfried Geyer

**Affiliations:** 1 Medical Sociology Unit, Hannover Medical School, Hannover, Germany; 2 Medical Psychology Unit, Hannover Medical School, Hannover, Germany

**Keywords:** Endocrinology, Health care

## Abstract

While socioeconomic inequalities in the prevalence and management of type 2 diabetes (T2D) are well established, little is known about whether inequalities exist in the prevalence and the temporal development of T2D comorbidities. Previous research points towards expansion of morbidity in T2D as depicted mainly by a rising trend of T2D comorbidities. Against this background, and using German claims data, this study aims to examine whether socioeconomic status (SES) inequalities exist in the rates and the temporal development of T2D comorbidities. Since previous research indicates varying risk levels for T2D prevalence in the population subgroups: working individuals, nonworking spouses and pensioners, the analyses are stratified by these three population subgroups. The study is done on a large population of statutory insured individuals with T2D in three time-periods between 2005 and 2017. Predicted probabilities of three comorbidity groups and the number of comorbidities were estimated using logistic and ordinal regression analyses among different income, education and occupation groups. Interaction analyses were applied to examine whether potential SES inequalities changed over time. The study showed that neither the cross-sectional existence, nor the temporal development of T2D comorbidities differed significantly among SES groups, ruling out SES inequalities in the prevalence and the temporal development of T2D comorbidities in Germany. In men and women of all examined population subgroups, predicted probabilities for less severe cardiovascular (CVD) comorbidities, other vascular diseases and the number of comorbidities per individual rose significantly over time regardless of SES, but little if any change took place for more severe CVD comorbidities. Another important finding is that the population subgroup of nonworking spouses had markedly higher predicted probabilities for most of the examined outcomes compared to working individuals. The study indicates that the expansion of morbidity in T2D in Germany does not appear to be SES-dependent, and applies equally to different population subgroups. Yet, the study highlights that nonworking spouses are a susceptible population subgroup that needs to be focused upon when planning and implementing T2D management interventions.

## Introduction

Type 2 Diabetes (T2D) is a rising global epidemic. Worldwide, 537 million individuals aged 20 -79 years have been reported to be diagnosed with diabetes, with an expected increase of 46% by the year 2045^1^. The high prevalence and associated burden of T2D makes this disease a major contributor to morbidity development, which follows one of three hypothesized paths. An optimistic hypothesis was proposed in the early 1980s by Fries, who suggested that better living conditions and improved primary prevention might delay the onset of disease and disability, leading to *morbidity compression*^2^. Gruenberg proposed an opposing hypothesis claiming that progress in medicine delays mortality leading to *morbidity expansion* due to more years lived with chronic disease^3^. The third hypothesis was formulated in 1982 by Manton who proposes a more dynamic pattern of morbidity development. His hypothesis, labeled as dynamic equilibrium, suggests that even with increasing disease rates and life expectancy, medical progress is associated with better disease management and quality of life, creating a state of equilibrium in morbidity development^4^.

In Germany, previous research examined the development of morbidity in the context of T2D. Besides the rise in the prevalence of T2D over time^5^, an earlier onset of T2D has been reported for younger age groups^6^. Simultaneously, life expectancy for individuals with T2D has been increasing, leading to more years lived with this chronic disease^5^, ruling out morbidity compression. To determine whether morbidity expansion or dynamic equilibrium applies in T2D, previous research examined the development of comorbidities in individuals with T2D using health insurance claims data. The study showed that among three examined age groups and both genders, there was a significant increase in the predicted probabilities of most T2D concordant comorbidities between 2005 and 2017^7^. It can therefore be assumed that the quality of life of individuals with T2D is rather worsening over time due to more comorbidities, confirming the hypothesis of morbidity expansion in this population.

An important predictor of T2D has been the socioeconomic status (SES), where studies from Europe report inequalities in favor of high SES groups as determined by education, income and occupation^8,9^. Despite improvements in the provision of health care and primary prevention, SES inequalities in T2D remain common and have even been reported to be increasing over time in several European countries^10–12^. Moreover, T2D is largely a lifestyle-dependent disease, where self-management abilities play an important role in determining the extent to which related cardiovascular and neuropathological complications are developed. Educational attainment might be most relevant for diabetes self-management; however, income and occupation determinants might also be important^8^. SES inequalities have also been reported in access to and utilization of diabetes care^13,14^. This implies that socioeconomic disparities would also be present in diabetes-related complications which are associated with inadequate glycemic control. While plenty of studies exist on SES inequalities in T2D, evidence on SES inequalities in T2D comorbidities is less abundant, with most studies originating from the UK and North America^15^. A few German studies examined SES inequalities in T2D long-term complications with somewhat contradicting results. A German study based on data from 1997 reported SES inequalities in glycated hemoglobin levels, but ruled out inequalities in other T2D comorbidities such as myocardial infarction, stroke and other cardiovascular (CVD) risk factors^16^. However, a recent study reported no SES differences in glycemic control in individuals with T2D one year after diagnosis^17^. Other studies focused on specific subgroups of individuals with T2D, such as those with diabetic nephropathy, where it was shown that SES inequalities exist in kidney function^18^.

The above-described studies from Germany are cross-sectional, and to our knowledge, no longitudinal studies exist on SES inequalities in T2D comorbidities. The fact that previous research points towards morbidity expansion in T2D in Germany^5–7^ makes it essential to not only examine whether SES inequalities exist in comorbidities, but also to understand whether potential inequalities are changing over time, and if so, in what direction. This would serve as a possible explanation for morbidity expansion and would help define subgroups where diabetes management programs should be particularly targeted at. Moreover, research suggests that different population subgroups have varying risk levels for morbidity and mortality, possibly due to different demography, lifestyle, exposure to risk factors and social roles that guide their health behavior^19,20^. Our previous research indicated that the population subgroup of non-working spouses is a vulnerable group when it comes to T2D prevalence. T2D was four times higher in male nonworking spouses and 2.6 times higher in female non-working spouses compared to male and female working individuals, respectively^8^. Therefore, this study aims to examine socioeconomic inequalities in the development of T2D concordant comorbidities over time in the three population subgroups: Working individuals, nonworking spouses and pensioners (other population subgroups are not considered due to data limitation reasons). Since income, education and occupation are three main socioeconomic indicators that may yet differ in the way and the extent to which they affect health^21–23^, this study considers the three indicators, with a focus on income due to better data comprehensiveness.

With respect to the above-mentioned considerations, and using German claims data of a large statutory health insurance provider available for the years 2005 to 2017, this study aims to investigate the following research questions:

1. Do SES inequalities exist in T2D-related comorbidities in three time periods between 2005 and 2017?
2. How are SES inequalities in T2D comorbidities developing over time between 2005 and 2017?
3. How does this differ among men and women of the three population subgroups: working individuals, nonworking spouses and pensioners?

## Methods

### Data

Health insurance in Germany is mandatory. Almost 90% of the population are statutory insured, with insurance premiums that depend on income^24^. One of the largest statutory health insurance providers in Germany is the “Allgemeine Ortskrankenkasse” (AOK). The AOK branch located in the federal state of Lower Saxony, the “Allgemeine Ortskrankenkasse Niedersachsen” (AOKN), insures almost one-third of the individuals living in this state and is the database used in this study. Previous research has shown that the AOKN population corresponds to the German population in terms of age and gender, while individuals of high income and occupational status are underrepresented^25,26^. Data provided by the AOKN are mainly collected for accounting purposes. The database includes demographic information, in- and outpatient diagnoses, medicinal prescriptions and undergone medical services.

This study did not require ethical approval, as it involved a pre-existing claims dataset created as part of the routine administrative activities of AOKN. Its scientific use is regulated by German law in the German Social Code “Sozialgesetzbuch”. The data protection officer of the Local Statutory Health Insurance of AOKN has given permission for this study to use the data for scientific purposes.

### Definition of T2D and comorbidities

The population of this study consists of AOKN-insured individuals with T2D aged 18 years and older. Diagnoses in AOKN are coded according to the 10^th^ version of the international classification of diseases (ICD-10). Since the database of this study is secondary, potential coding errors could lead to inaccuracies in the definition of T2D and comorbidities. Thus, defining T2D as well as comorbidities was done according to certain criteria and similar to a previous study^7^. Confirmed outpatient diagnoses and primary and secondary inpatient diagnoses were considered for the definition of T2D cases as well as the chronic comorbidities such as hypertension. Outpatient diagnoses with the data fields “suspected” or “ruled out” were not considered for T2D diagnoses as well as the comorbidities depending on the type of comorbidity. Diagnoses were only considered eligible if they were coded in two quarters of the observation period, with an exception for those who were insured during only one quarter.

Diabetes mellitus is assigned the ICD-10 codes E10-E14, with E11 referring to T2D. However, inaccurate or double coding were present in the data in some cases. Therefore, the definition of T2D cases was done according the following criteria: First, when E11 was the most frequently coded diagnoses in the observation period (among other diabetes diagnoses), individuals were considered to have T2D. Second, since T2D represents around 90% of all diabetes cases, individuals were also considered to have T2D if E14 (which refers to the category “undefined”) was the most frequently coded diagnoses. If E10 (referring to the category “type 1 diabetes”) was most frequently coded but no insulin was prescribed, individuals were also considered to have T2D.

Comorbidities considered in this study were: hypertension, hyperlipidemia, cardiac insufficiency, angina pectoris, myocardial infarction, stroke, retinopathy, nephropathy and polyneuropathy which were chosen according to previous research^27,28^. Where it was not clear which codes correspond to the comorbidities in question, the definition of codes was done according to that of the Scientific Institute of AOK (WIdO)^29^. WIdO publishes consecutive reports on different diseases and the utilization of medical services using claims data of the AOK^30^. The list of ICD-10 codes as well as the types of diagnoses used to define the examined comorbidities is found in Supplementary Table S1.

Since the study considers the comorbidities as a measure of the state of morbidity in the T2D population, it is not relevant whether the comorbidities were diagnosed before or after T2D. Therefore, all individuals that matched the diagnosis criteria for T2D described above were included in the study.

### Time-period

Trends were examined over the three time-periods: 2005–2007 (p1), 2010–2012 (p2) and 2015–2017 (p3), illustrating equal intervals and gaps in-between. Using three time-periods with three-year intervals and two-year gaps in between over the whole period for which the data was available was optimal to clearly demonstrate the direction of temporal development of comorbidities while providing enough time for noticeable developments in the outcomes to take place. Moreover, T2D cases as well as the comorbidities were defined newly in each period to allow for the same source of potential errors in the definition, thus enhancing the comparability of the time-periods.

### Population subgroups

The analyses in this study were performed separately for the three population subgroups: working individuals, non-working spouses and pensioners. In the database of AOKN, insured individuals are subdivided according to their insurance status. Employed individuals are the subgroup of people in paid employment who are liable for social insurance. Nonworking spouses are the subgroup of men and women whose insurance is covered under that of their employed spouses (family insurance), and are thus exempted from paying their own insurance premiums. They are distinguished from unemployed individuals in which they are financially covered by their employed spouses. The subgroup pensioners are retired men and women after reaching the age threshold (65+ years), with a small minority of early-pensioners who retired mostly for health-related reasons. Since the insurance status of individuals might change, population subgroup in this study was identified newly in each time-period according to the most frequently registered insurance status per period. Following up only individuals who had the same insurance status at the three periods was avoided since this might lead to biased results. This is because this way, we would be following up the same individuals who are getting older and having more time after diagnoses behind them, which would naturally lead to more comorbidities.

### Socioeconomic indicators

In claims data of the AOKN, information on income, education and occupation of insured individuals are provided by the employers. Thus, socioeconomic information in the data is only available for employed individuals. One exception is the availability of income information for pensioners, which is provided by the German Pension Insurance. This implies that SES information is not directly available for the subgroup nonworking spouses.

This study focuses on income because it is the only SES indicator available for pensioners, a very important population subgroup when investigating T2D complications and SES disparities due to the higher age range. Inequalities in education and occupation in employed individuals and nonworking spouses were additionally investigated for argument and conclusion affirmation purposes, and corresponding results are provided comprehensively in the supplementary files.

Since no SES information was directly available for the subgroup of nonworking spouses, information on income, education and occupation was transformed from their employed spouses. While some limitations are associated with this procedure, transforming spouse or family SES information has been a common practice in scientific research^31–33^. This practice has been supported by the argument of homogamy, which suggests that partners are likely to have similar social backgrounds. Moreover, research also suggests that partners are usually exposed to similar health risks or protective factors due to sharing a similar social environment. Thus, under the assumption of homogamy, assigning spouses’ SES can be the best solution scenario when individual SES information is missing for important target groups, in this case non-working spouses who were shown to have a higher risk for T2D^8^.

*Income* is classified in AOKN according to the proportion of the German average annual income (AGI) for the former Western federal states of Germany in terms of the pretax salary of employed individuals as reported yearly by the Federal Statistical Office of Germany (Statistisches Bundesamt). To make the income of pensioners more comparable to the average German annual salary of working individuals, the AGI was adjusted for unemployment insurance and pension insurance contributions that all employed individuals pay as part of their taxes. The proportions of these contributions differ after individuals enter the pension. In this study, for each year of observation, the corresponding adjusted AGI served as a reference, and income was classified as the deviation from the yearly AGI. Individual income per insured person was classified into three levels labeled as low (<60% of the AGI), middle (60–80% of the AGI) and higher (>80 % of the AGI). For each of the three time-periods, the highest income level of the three years within each period was considered. The reference income figures as well as the respective mean numeric income for the years 2005 to 2017 for the three income levels are found in Supplementary Table S2. Several reasons apply in favor of this solution: By using annual averages, it is possible to compare social gradients over analyses with different diseases. If the income distribution or the range of a particular study sample or a study population does not correspond to the population distribution, the cut-off-values are unique to this study population, thus making comparisons between studies impossible. As the social structure of the AOKN does not correspond to the structure of Germany^26^, the respective income-cut-offs may not correspond to the national figures. Moreover, since the income distribution of the AOKN population differs from that of the general average income in terms of high income groups being underrepresented in AOKN^26^, setting higher limits for the higher income group would lead to having a skewed distribution. Therefore, the cut off for “higher” income was set to >80% of the AGI.

*Education* is classified according to the highest achieved school leaving certificate and displayed as the years of schooling with the three levels: ≤9 years of schooling (German Hauptschulabschluss or no school diploma which applies to only 2% of the study population), 10 years of schooling (German Realschulabschluss) and 12–13 years of schooling (German Abitur which is equivalent to a high school diploma).

*Occupation* in the data is classified originally according to the German classification of occupations as provided by the Federal Employment Agency (Bundesagentur für Arbeit). The very detailed classifications were then summarized into 12 groups according to the occupation classification system developed by Blossfeld^34^. The 12 groups were then summarized into three levels based on qualification level and task complexity as follows: Manuals, specialists and highly qualified.

### Statistical analyses

Statistical analyses in this study were stratified by gender and the three population subgroups: working individuals, nonworking spouses and pensioners, creating up to six subgroups per analysis.

The temporal development of comorbidities was investigated and compared among the different SES groups within the examined subgroups. In pensioners, the development of comorbidities was examined and compared only among the three income groups since the data lacks information on occupation and education for this population subgroup.

#### Development of comorbidity groups

This study focused on nine common T2D concordant comorbidities chosen after a thorough literature review. Since the study involves several layers of analyses due to comparing across population subgroups, SES and gender, the nine comorbidities were grouped into three outcomes to provide more clarity and an overview of the results. The three comorbidity groups are *less severe CVD comorbidities* (having at least one of the comorbidities: hypertension, hyperlipidemia and cardiac insufficiency), *more severe CVD comorbidities* (having at least one of the comorbidities: Stroke, myocardial infarction and angina pectoris) and *other vascular diseases* (having at least one of the comorbidities: retinopathy, nephropathy and polyneuropathy). The development of comorbidity groups across the different SES levels was examined by means of logistic regression analysis. The dependent variable was the comorbidity group, and the main independent variable was the time-period, with p1 being the reference group. A separate logistic regression analysis was applied for each gender, population subgroup and SES level. In all models, cluster robust standard errors were used to adjust for within-cluster variation. This was essential in order to correct for autocorrelation associated with having some individuals in more than one time-period. Age and duration of observation were adjusted for in all models to correct for unequal observation periods, since insured individuals are not all observed for the same amount of time per period. For each SES indicators, margins at the mean age of the three corresponding SES groups were used in order to allow for an age-standardized comparison across the SES groups and to rule out any age-related effects. Results are presented in terms of predicted probabilities (figures) and prevalence ratios (PR) (tables). PRs are more adequate than odds ratios when outcomes have a prevalence rate of higher than 10%, which is the case for most comorbidities considered in this study^7^. For outcomes with higher prevalence rates, odds ratios display more extreme effect sizes by either overestimating (when OR>1) or underestimating (when OR<1) effects.

#### Development of the number of comorbidities

The outcome number of comorbidities was also used to illustrate the temporal development of comorbidities. The number of comorbidities per individual was grouped into four categories: (0, 1, 2 and >2). Ordinal logistic regression analyses were applied to examine the effect of time-period (p2 or p3 compared to p1) on the chance of being one category higher in the outcome—i.e. having at least one additional comorbidity. Ordinal logistic regression is a form of regression analysis where the outcome variable is measured at an ordinal level. It allows the accurate modeling of an outcome with ordinal response categories, as is the case in this analysis, which can improve model accuracy. The odds ratios as the main results of ordinal logistic regression are interpreted as the relative change in odds of being at least one category higher in the outcome variable—in this case having at least one additional comorbidity. All 46 models adjusted for age and duration of observation and used robust standard errors to correct for possible autocorrelation. Here too, margins were used at the mean age of the different SES levels per corresponding SES indicator to rule out age effects. Results were displayed in terms of predicted probabilities (figures) and ORs (tables).

#### Temporal change in SES inequalities

Besides the descriptive comparison that was made available by the above-described statistical analyses, the temporal change in SES inequalities was examined by interaction analyses via logistic regression. One logistic regression model was applied per population subgroup, gender, and SES indicator (i.e., 14 models per outcome). Two-way interaction terms of time-period and SES indicators were included in the models while adjusting for age and duration of observation and using robust standard errors to correct for autocorrelation. The reference categories were p1 and the lowest level of each SES indicator. Significant interactions imply socioeconomic inequalities in the temporal development of comorbidities in T2D. Since the database of this study is a whole population with large N, a p<0.05 is easily obtained. Therefore, stricter criteria were followed to consider results as “significant”. Results are considered significant at p<0.001, when confidence intervals do not include one, and when the significant interaction is also observed at p3, and not just at p2.

### Ethical approval

This study did not require ethical approval. The analyses were performed using a pre-existing claims dataset created as part of the routine administrative activities of a statutory health insurance provider. Its scientific use is regulated by German law in the German Social Code “Sozialgesetzbuch”. The data protection officer of the Local Statutory Health Insurance of Lower Saxony-AOK Niedersachsen (Germany) has given permission for this study to use the data for scientific purposes. The study was conducted in accordance with the Declaration of Helsinki.

### Informed consent statement

Informed consent was not needed since the database in this study is a pre-existing anonymized claims dataset and contact to patients did not exist in any form.

## Results

At p1, the study population consisted of 226124 individuals with T2D. At p2, 101975 ‬individuals from the T2D population at p1 left the AOKN, and 153065 individuals joined and matched the inclusion criteria, consisting a population of 277214 individuals at p2. The population at p3 consisted of 283468 individuals from which 92706 individuals were present at p2 and 87076 individuals were present at p1. Characteristics of the study population stratified by population subgroup, gender and time-period are represented in Table 1.

**Table 1:** Population characteristics stratified by population subgroup and time-period.

	Working individuals			Non-working Spouses			Pensioners		
	p1	p2	p3	p1	p2	p3	p1	p2	p3
N	28956	43958	53495	16435	20003	15609	180733	213253	214364
Age mean (SD)	52 (9)	53 (9)	54 (9)	57 (11)	58 (12)	59 (12)	74 (10)	75 (9)	76 (10)
Gender n (%)									
Men	19978 (69%)	29994 (68%)	35268 (66%)	2494 (15%)	3517 (18%)	3069 (20%)	73441 (41%)	91992 (43%)	95242 (44%)
Women	8978 (31%)	13964 (32%)	18227 (34%)	13941 (85%)	16486 (82%)	12540 (80%)	107292 (59%)	121261 (57%)	119122 (56%)
Insurance duration *days* mean (SD)	1048 (165)	1045 (159)	1037 (194)	1033 (195)	1035 (185)	1029 (207)	1001 (241)	994 (241)	992 (254)
Income n (%)									
Low	6187 (21%)	10867 (25%)	14163 (27%)	5328 (32%)	6719 (34%)	5094 (33%)	130122 (72%)	155366 (73%)	162156 (76%)
Middle	3717 (13%)	6493 (15%)	9270 (17%)	2578 (16%)	2848 (14%)	2421 (15%)	35670 (20%)	37784 (18%)	34764 (16%)
Higher	19052 (66%)	26598 (60%)	30062 (56%)	8529 (52%)	10436 (52%)	8094 (52%)	14941 (8%)	20103 (9%)	17444 (8%)
Education (years of schooling) n (%)									
≤ 9 years	6350 (64%)	15411 (61%)	18533 (54%)	1885 (66%)	3518 (66%)	3665 (63%)	–	–	–
10 years	2992 (30%)	8131 (32%)	12412 (36%)	812 (28%)	1523 (28%)	1721 (30%)	–	–	–
12–13 years	621 (6%)	1777 (7%)	3081 (9%)	156 (6%)	325 (6%)	380 (7%)	–	–	–
Occupation n (%)									
Manuals	21918 (84%)	31591 (78%)	38340 (77%)	5889 (90%)	7740 (87%)	6748 (85%)	–	–	–
Specialists	3583 (14%)	7031 (17%)	8653 (17%)	548 (8%)	893 (10%)	889 (11%)	–	–	–
Highly qualified	509 (2%)	1827 (5%)	2611 (5%)	101 (2%)	302 (3%)	342 (4%)	–	–	–
Comorbidities n (%)									
Less severe CVD	19702 (68%)	32744 (75%)	42976 (80%)	12444 (76%)	16114 (81%)	13358 (86%)	159654 (88%)	198019 (93%)	204736 (96%)
More severe CVD	1559 (5%)	2348 (5%)	2696 (5%)	1135 (7%)	1438 (7%)	1079 (7%)	24196 (13%)	27481 (13%)	25102 (12%)
Other vascular diseases	3571 (12%)	7242 (17%)	11507 (22%)	3194 (19%)	5140 (26%)	5180 (33%)	53382 (30%)	84833 (40%)	111198 (52%)
0 Comorbidity	7931 (27%)	9511 (22%)	8428 (16%)	3217 (20%)	3106 (16%)	1677 (11%)	13414 (7%)	9420 (4%)	4917 (2%)
1 Comorbidity	10552 (36%)	15196 (35%)	16687 (31%)	5536 (34%)	6105 (30%)	3962 (25%)	42735 (24%)	39043 (18%)	24870 (12%)
2 Comorbidities	7134 (25%)	12470 (28%)	16107 (30%)	4530 (27%)	5614 (28%)	4323 (28%)	55265 (31%)	61154 (29%)	50312 (23%)
>2 Comorbidities	3339 (12%)	6781 (15%)	12273 (23%)	3152 (19%)	5178 (26%)	5647 (36%)	69319 (38%)	103636 (49%)	134265 (63%)

*Time- periods* p1: 2005–2007, p2: 2010–2012, p3:2015–2017. *Comorbidities* Less severe CVD: Hypertension, Hyperlipidemia, Cardiac insufficiency; More severe CVD: Myocardial infarction, Stroke, Angina Pectoris; Other vascular diseases: Nephropathy, Neuropathy, Retinopathy. *Income* Low: <60% AGI, Middle: 60–80% AGI, Higher: >80% AGI.

### Income

**Figure 1:**
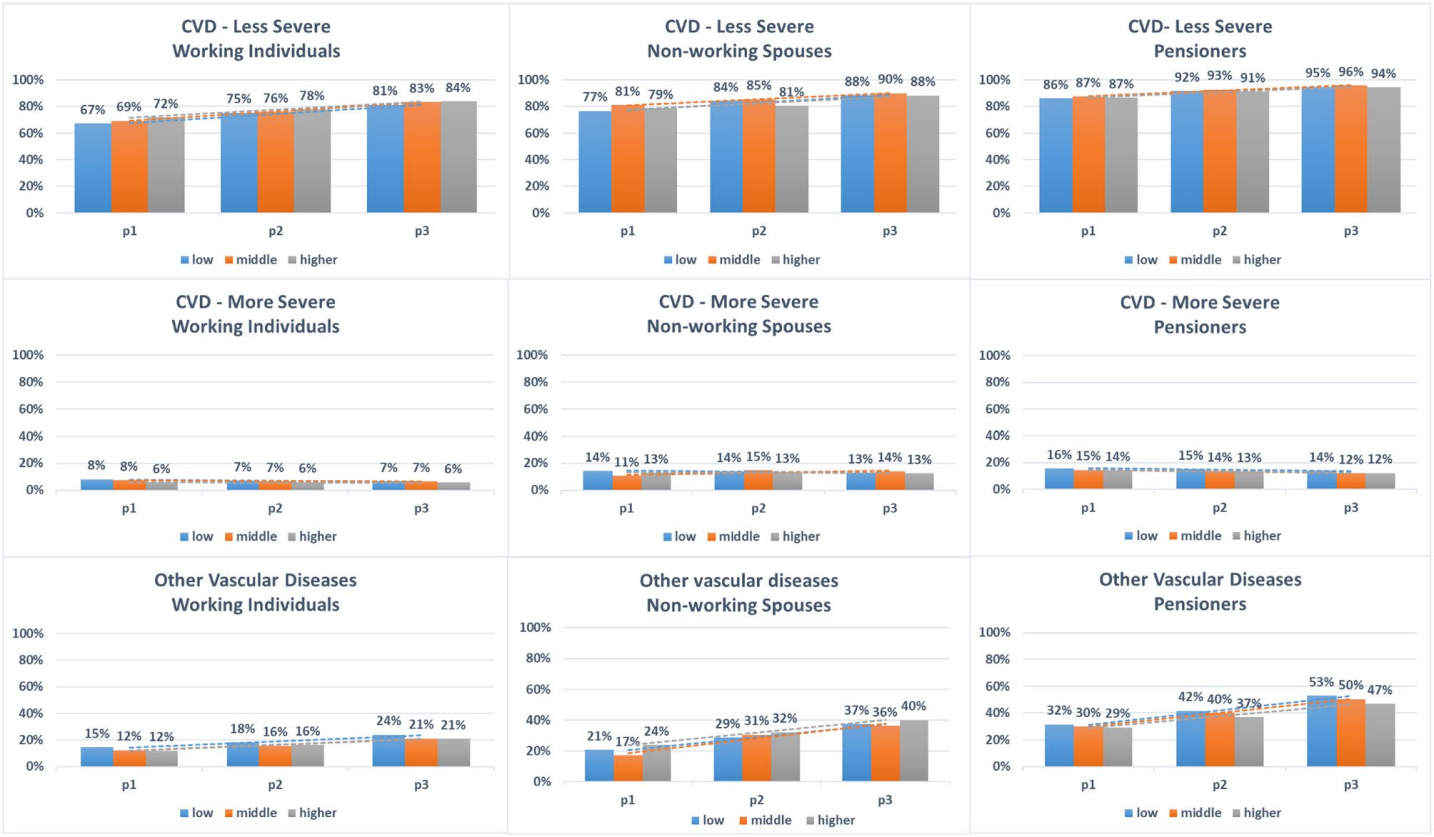
Predicted probabilities of the three comorbidities groups over the three time-periods for **men**, stratified by income and population subgroup. *Time-periods* p1: 2005–2007, p2: 2010–2012, p3:2015–2017. *Comorbidities* Less severe CVD: Hypertension, Hyperlipidemia, Cardiac insufficiency; More severe CVD: Myocardial infarction, Stroke, Angina Pectoris; Other vascular diseases: Nephropathy, Neuropathy, Retinopathy. *Income* Low: <60% AGI, Middle: 60–80% AGI, Higher: >80% AGI.

**Figure 2:**
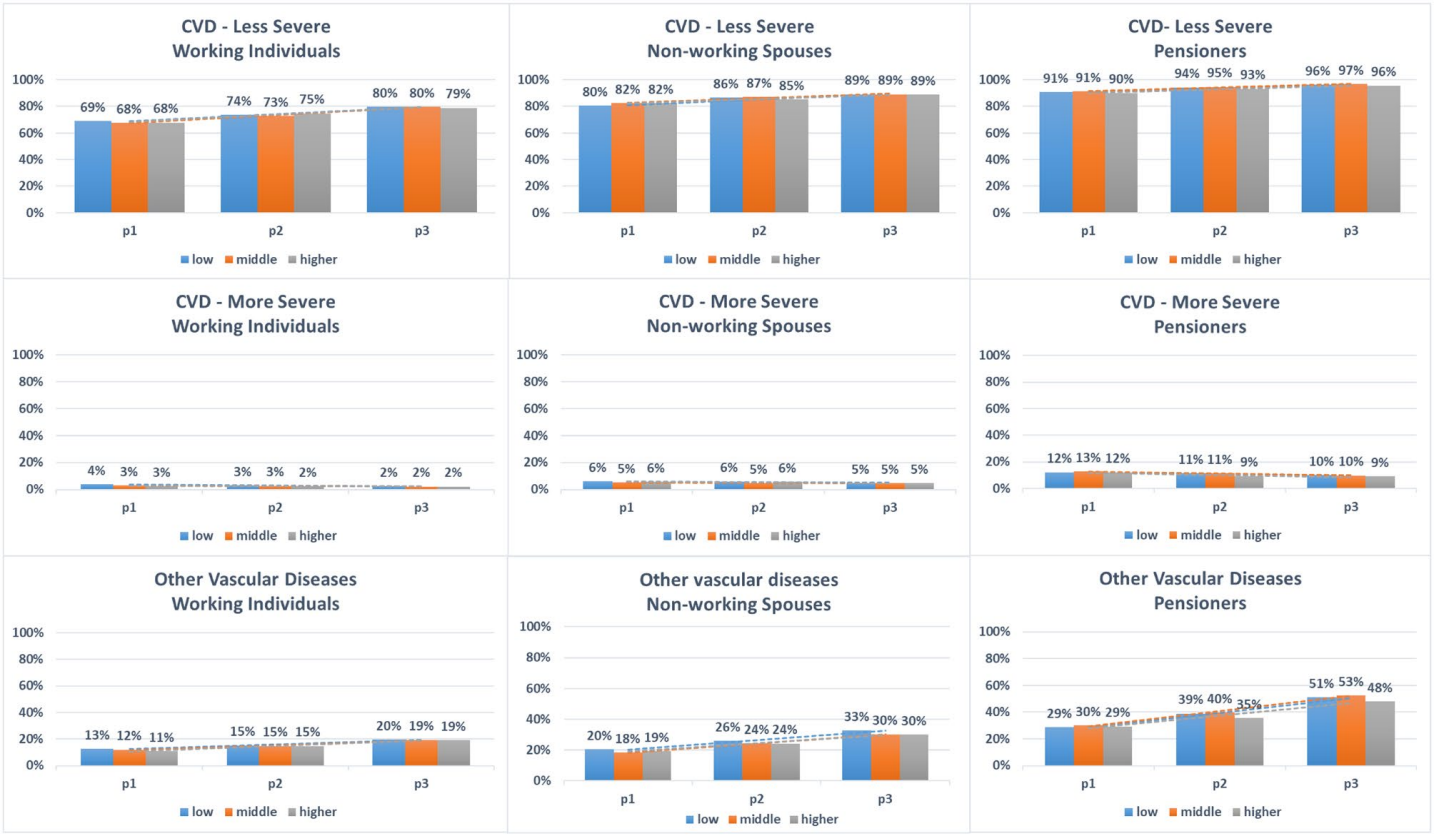
Predicted probabilities of the three comorbidities groups over the three time-periods for **women**, stratified by income and population subgroup. *Time-periods* p1: 2005–2007, p2: 2010–2012, p3:2015–2017. *Comorbidities* Less severe CVD: Hypertension, Hyperlipidemia, Cardiac insufficiency; More severe CVD: Myocardial infarction, Stroke, Angina Pectoris; Other vascular diseases: Nephropathy, Neuropathy, Retinopathy. *Income* Low: <60% AGI, Middle: 60–80% AGI, Higher: >80% AGI.

For less severe CVD comorbidities (Hypertension, hyperlipidemia and cardiac insufficiency), the predicted probabilities increased markedly and significantly for men and women in all three population subgroups. Almost two-thirds of working individuals, three-quarters of non-working spouses and over 86% of pensioners had less severe comorbidities in p1. The predicted probabilities increased by more than 10% points in p3 for all subgroups examined. Similarly, there was a marked increase in the predicted probabilities for other vascular diseases in men and women among all population subgroups. 11% to 15% of working individuals had comorbidities of this group in p1, and the predicted probabilities increased by up to 10% points in p3. In nonworking spouses, the predicted probabilities for other vascular diseases were markedly higher compared to working individuals and the temporal increase was more pronounced, with 37-40% of men having other vascular diseases in p3 compared to 17–24% in p1. However, the increase in predicted probabilities in nonworking spouses was less pronounced in women compared to men. The increase in predicted probabilities for other vascular diseases was also observed for female and male pensioners, with almost half of pensioners having comorbidities of this group in p3 compared to around one-third in p1 (Figs. 1, 2 and Table 2).

**Table 2:** Prevalence/odds ratios and confidence intervals on the effect of time-period on the 3 comorbidity-index variables and the number of comorbidities, stratified by gender, population subgroup and income group.

	n	Less severe CVD CMs				More severe CVD CMs				Other vascular diseases				Number of comorbidities			
		p2		p3		p2		p3		p2		p3		p2		p3	
		PR	95% CI	PR	95% CI	PR	95% CI	PR	95% CI	PR	95% CI	PR	95% CI	OR	95% CI	OR	95% CI
Men																	
Employed																	
Low income	12064	1.12	1.08–1.16	1.21	1.17–1.25	0.87	0.73–1.01	0.82	0.69–0.95	1.23	1.12–1.39	1.64	1.47–1.81	1.35	1.23–1.47	2.09	1.91–2.87
Middle income	11893	1.1	1.06–1.13	1.2	1.16–1.24	0.86	0.69–1.02	0.89	0.72–1.05	1.26	1.08–1.43	1.7	1.48–1.92	1.36	1.23–1.49	2.15	1.95–2.36
Higher income	61283	1.08	1.07–1.1	1.17	1.15–1.18	1.02	0.94–1.1	0.96	0.88–1.04	1.36	1.29–1.42	1.75	1.67–1.84	1.4	1.35–1.45	2.13	2.05–2.21
Non-working spouses																	
Low income	3313	1.1	1.05–1.15	1.15	1.1–1.2	0.97	0.77–1.17	0.9	0.7–1.1	1.39	1.18–1.59	1.82	1.54–2.09	1.53	1.32–1.77	2.37	2–2.81
Middle income	732	1.05	0.97–1.13	1.11	1.03–1.19	1.38	0.69–2.06	1.32	0.66–1.98	1.77	1.16–2.39	2.11	1.36–2.85	1.77	1.29–2.42	2.4	1.72–3.35
Higher income	5035	1.02	0.99–1.06	1.12	1.08–1.16	1.02	0.84–1.2	0.96	0.78–1.15	1.34	1.2–1.49	1.67	1.48–1.87	1.26	1.13–1.42	2.07	1.81–2.37
Pensioners																	
Low income	166456	1.06	106–1.07	1.1	1.09–1.1	0.96	0.93–0.99	0.88	0.85–0.9	1.32	1.3–1.34	1.68	1.65–1.7	1.6	1.56–1.63	2.8	2.74–2.87
Middle income	65285	1.06	1.06–1.07	1.09	1.09–1.1	0.95	0.91–0.99	0.82	0.78–0.86	1.33	1.3–1.36	1.67	1.63–1.72	1.59	1.54–1.64	2.91	2.81–3.03
Higher income	28934	1.05	1.04–1.06	1.09	1.08–1.1	0.93	0.87–1	0.85	0.79–0.91	1.26	1.21–1.3	1.59	1.53–1.65	1.44	1.37–1.51	2.5	2.37–2.64
Women																	
Employed																	
Low income	19153	1.07	1.04–1.09	1.15	1.12–1.18	0.82	0.66–0.98	0.65	0.52–0.78	1.21	1.09–1.32	1.55	1.40–1.69	1.25	1.17–1.34	1.89	1.76–2.03
Middle income	7585	1.08	1.03–1.12	1.18	1.13–1.22	0.95	0.62–1.27	0.79	0.52–1.05	1.23	1.05–1.42	1.62	1.39–1.86	1.26	1.14–1.40	1.98	1.78–2.21
Higher income	14429	1.1	1.07–1.13	1.16	1.13–1.19	0.96	0.70–1.2	0.89	0.67–1.13	1.36	1.21–1.51	1.76	1.57–1.95	1.42	1.25–1.62	1.94	1.71–2.2
Non-working Spouses																	
Low income	13828	1.07	1.06–1.09	1.11	1.09–1.13	0.98	0.85–1.12	0.8	0.67–0.93	1.28	1.2–1.37	1.61	1.49–1.77	1.43	1.33–1.52	2.07	1.91–2.24
Middle income	7115	1.06	1.03–1.08	1.08	1.06–1.11	0.86	0.68–1.05	0.9	0.68–1.11	1.32	1.19–1.45	1.64	1.47–181	1.38	1.26–1.52	1.98	1.78–2.21
Higher income	22024	1.03	1.02–1.04	1.08	1.06–1.09	0.98	0.85–1.11	0.8	0.67–0.92	1.26	1.18–1.34	1.57	1.47–1.67	1.26	1.19–1.32	1.91	1.8–2.04
Pensioners																	
Low income	281188	1.04	1.04–1.04	1.06	1.06–1.06	0.95	0.93–0.97	0.82	0.8–0.84	1.36	1.34–1.37	1.78	1.76–1.8	1.5	1.48–1.52	2.62	2.57–2.66
Middle income	42933	1.04	1.03–1.04	1.06	1.06–1.07	0.89	0.84–0.95	0.78	0.73–0.83	1.32	1.29–1.36	1.75	1.70–1.81	1.44	1.38–1.49	2.56	2.45–2.68
Higher income	23554	1.04	1.03–1.05	1.06	1.05–1.07	0.77	0.7–0.85	0.74	0.67–0.82	1.21	1.15–1.26	1.63	1.56–1.71	1.33	1.26–1.41	2.09	2.15–2.44

Estimated by means of logistic regression and ordinal regression, adjusting for within cluster variation. Adjusted for age and insurance duration. *Time-periods* p1: 2005–2007, p2: 2010–2012, p3:2015-2017. *Comorbidities* Less severe CVD: Hypertension, Hyperlipidemia, Cardiac insufficiency; More severe CVD: Myocardial infarction, Stroke, Angina Pectoris; Other vascular diseases: Nephropathy, Neuropathy, Retinopathy. *Income* Low: <60% AGI, Middle: 60–80% AGI, Higher: >80% AGI.

The predicted probabilities for more severe CVD remained constant for most of the examined subgroups, while there was a temporal decrease in predicted probabilities in other subgroups. The only pronounced temporal reduction was observed for the subgroup: working women with low income, where the predicted probabilities halved between p1 and p3. For this comorbidity group, the predicted probabilities were almost double as high for nonworking spouses compared to working individuals. In male nonworking spouses, predicted probabilities were almost similar to those of male pensioners (Figs. 1, 2 and Table 2).

**Figure 3:**
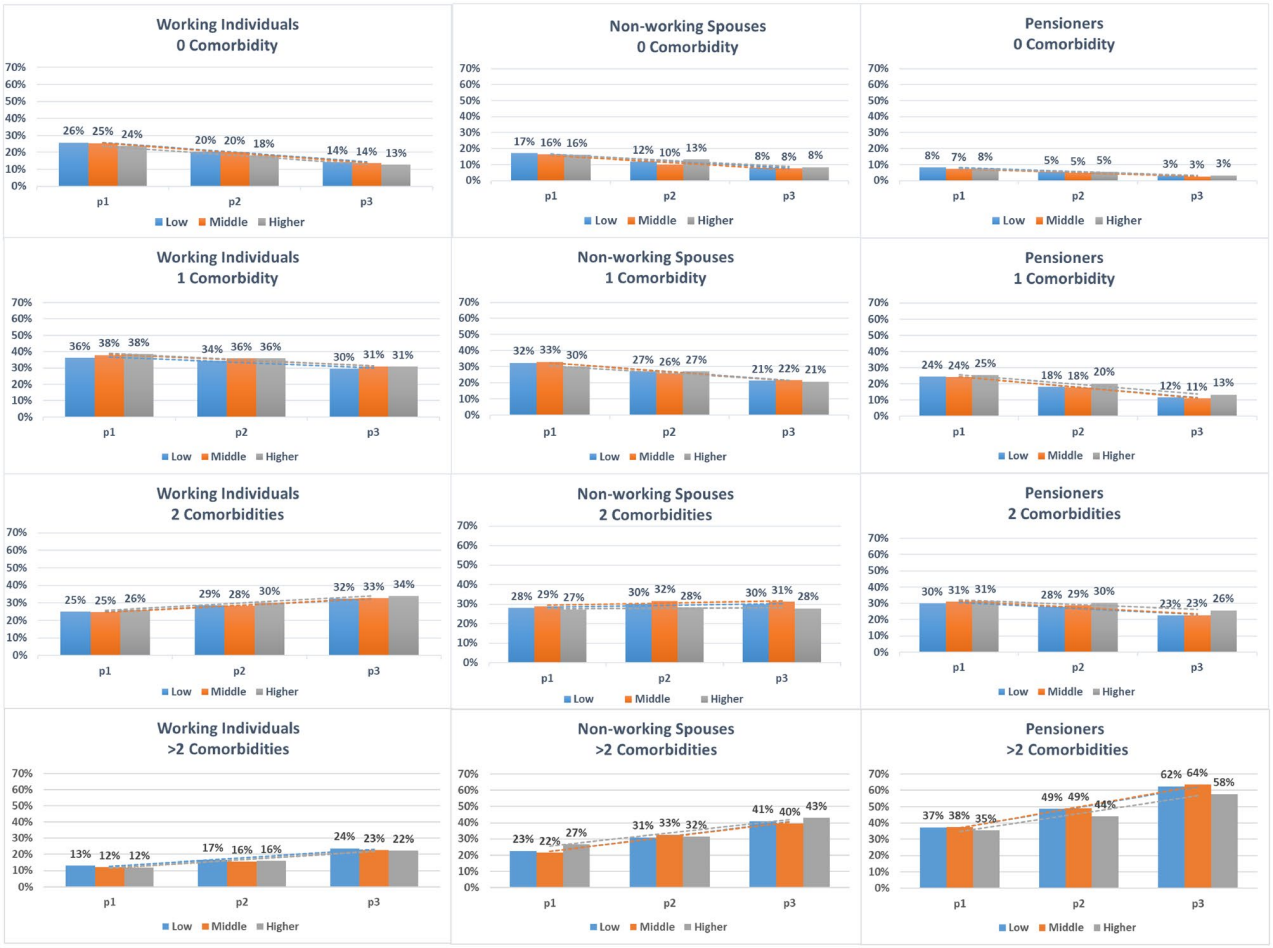
Predicted probabilities of the number of comorbidities over the three time-periods for men, stratified by income and population subgroup. *Time-periods* p1: 2005–2007, p2: 2010–2012, p3:2015–2017. *Income* Low: <60% AGI, Middle: 60–80% AGI, Higher: >80% AGI.

**Figure 4:**
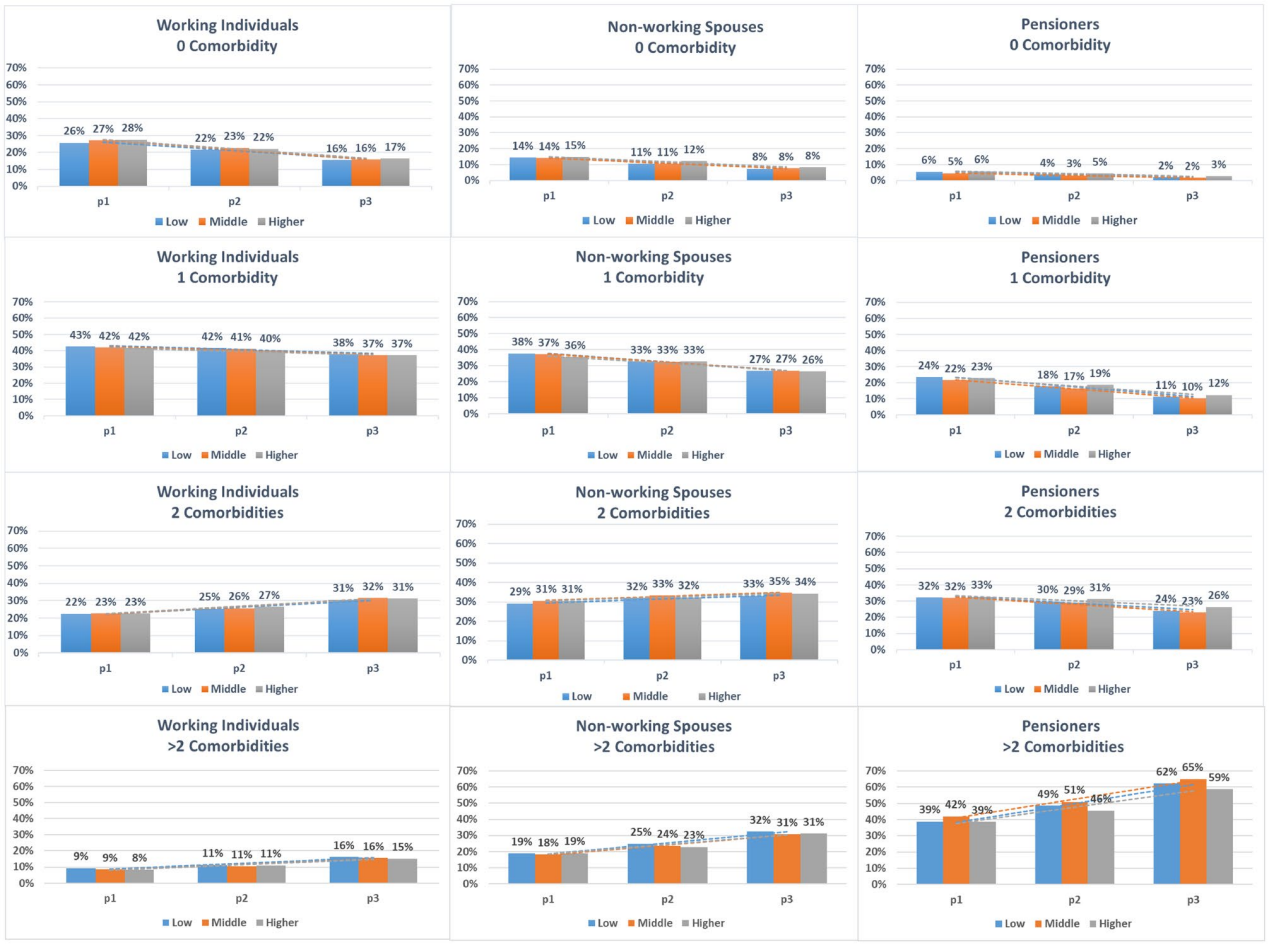
Predicted probabilities of the number of comorbidities over the three time-periods for women, stratified by income and population subgroup. *Time-periods* p1: 2005–2007, p2: 2010–2012, p3:2015–2017. *Income* Low: <60% AGI, Middle: 60–80% AGI, Higher: >80% AGI.

A consistent increase in predicted probabilities was also observed for the number of comorbidities in all examined subgroups (Figs. 3, 4 and Table 2). Nevertheless, in nonworking spouses (both genders), the predicted probabilities of having more than two comorbidities was almost double that of working individuals, which also consistently increased over time.

The predicted probabilities did not differ substantially among the three income groups in both genders and the three examined population subgroups. The difference between income groups was only a few percentage points if any, which applies for the three outcomes and all examined subgroups.

The interaction analyses also revealed that there were no significant interactions between time-period and income for working individuals and nonworking spouses. In female pensioners, the interaction analysis revealed a tendency towards socioeconomic inequalities in the comorbidity group ‘other vascular diseases’. This was also observed for the outcome ‘number of comorbidities’, where both males and females had a tendency towards income inequalities illustrated with a more pronounced morbidity expansion (or increase in the predicted probabilities) in the lower income compared to the higher income group (Figs. 1, 2 & Supplementary Tables S3 to S6).

### Education and occupation

A consistent increase in predicted probabilities could also be observed when stratifying by education (Supplementary Figs. S1 to S4 and Supplementary Table S7) and occupation (Supplementary Figs. S5 to S8 and Supplementary Table S8). In working individuals, the three education groups illustrated a similar temporal increase in the predicted probabilities (Supplementary Figs. S1 to S4 and Supplementary Table S7), and no significant interactions were observed (Supplementary Tables S3 to S6). In nonworking spouses, comparing the development of predicted probabilities between the three education/occupation groups indicates a more pronounced increase in the school education group “≤ 9 years of schooling” or “manuals” compared to the school education group “12-13 years of schooling” or highly qualified groups, respectively, which was yet not consistent for all outcomes investigated (Supplementary Figs. S1 to S8). However, the interaction analysis did not show any significant interactions between time and education/occupation (Supplementary Tables S3 to S6), ruling out consistent education or occupation inequalities in the development of comorbidities in T2D.

## Discussion

This study investigated SES inequalities in the development of comorbidities in working individuals, nonworking spouses and pensioners. The analyses showed no clear differences in the development of comorbidities among different SES groups, which applied for the three SES indicators and the three population subgroups examined. Nevertheless, the study showed that the predicted probabilities for less severe CVD comorbidities and other vascular diseases increased significantly over the examined time-periods with a rising trend over time, which is in line with the results of our previous study^7^. This study however added that morbidity expansion applies even when stratifying the population into subgroups that differ in the state of employment and social roles as well as SES.

### SES inequalities in T2D comorbidities

SES differences in the existence and the temporal development of comorbidities has not been abundantly investigated. A German cross-sectional study indicated a similar conclusion on the lack of SES inequalities in the outcomes and risk factors of CVD in individuals with T2D, but pointed towards inequalities with respect to glycated hemoglobin^16^. A recent German study however ruled out an association between SES and level of glycated hemoglobin^17^. The lack of SES inequalities in T2D comorbidities can be explained by several arguments. The development of T2D comorbidities largely depends on disease management. Health literacy, self-efficacy and access to health care are major determinants of disease self-management that have been shown to be reliant on individual SES level^35–40^, especially education. Moreover, the level of education has been found to be the strongest predictor for T2D^8^, and it is speculated that this might be due to its mediating effect with respect to health literacy and access to health care. Thus, it was hypothesized that SES inequalities, particularly education, would exist in the development of T2D comorbidities. This study ruled out this hypothesis for the population of Lower Saxony in Germany. One possible explanation is that T2D has been reported to be less common in higher SES groups^8,41^. Individuals with high SES that have T2D might thus represent a very selective group that presumably differs from other individuals of high SES. Hence, it can be assumed that health-promoting behavior, health literacy, and rates of risk factors are less favorable in this subgroup. From this perspective, it seems understandable that probabilities for T2D comorbidities in higher SES did not differ from the other SES groups. Another possible explanation could lie behind the fact that T2D is often diagnosed at later stages, where comorbidities have already developed^42,43^. This can partly explain the high prevalence of comorbidities in the population of T2D, but also indicates that risk factors have already accumulated to an extent that outweighs the effect of SES after T2D had been diagnosed. A German study indicated that disability rates increased over time in individuals with T2D with obesity having a significant mediating effect^44^. In addition, T2D is strongly associated with obesity with evidence on a causal association and obesity-associated insulin resistance^45–47^. In addition, obesity is mainly a lifestyle-dependent condition with a clear link to individual and environmental SES^48^. This implies that SES inequalities might have already reached their threshold before the diagnoses of T2D, so that they no longer play a role afterwards. Nevertheless, it should be noted that most available studies beyond Germany show contradicting results with respect to SES inequalities in T2D comorbidities, especially for CVD comorbidities. Even among European studies, an association was observed between individual SES as well as geographic deprivation and risk for CVD complications^15^. The difference might lie within the quality of care and management in individuals with T2D. In Germany, health insurance is mandatory and insurance fees depend on income, with almost 90% of the population being statutory insured^24^. Thus, Germany is a universal welfare state where access to health care services and medical therapy is theoretically equal in all SES groups. For example, medical services such as disease management programs (DMPs) are equally offered for all concerned members of society. While there is no evidence on whether SES differences exist in the enrollment of diabetes specific DMPs, a German study examined whether education inequalities exist in the enrollment of DMPs in patients with coronary heart disease and found no significant association^49^. Thus, universal access to DMPs coupled with greater public awareness of T2D consequences could be limiting the existence of SES differences in T2D comorbidities. Nevertheless, it remains possible that individuals with a higher SES get more often diagnosed for diabetes and comorbidities due to having higher health literacy, which masks and balances potential differences in the prevalence of comorbidities between them and those with a lower SES.

### Trend of SES inequalities in T2D comorbidities

The lack of association between SES level and the risk of having T2D comorbidities remained to be largely consistent over time. Our previous research on the same population indicated an increase in the risk of less severe CVD comorbidities and other vascular diseases, as well as a majorly unchanging trend in more severe CVD comorbidities between 2005 and 2017^7^. These results were replicated in this study where it was shown that trends are similar among all SES groups, ruling out that temporal change in the SES structure of individuals with T2D would be a reason behind morbidity expansion in this population. Thus, morbidity expansion in T2D applies for all SES groups without any clear differences in its extent and no clearly observed gradients, which contradicts with theoretical expectations of socioeconomic inequalities^50,51^. While this clearly applied for the two population subgroups: working individuals and non-working spouses, the interaction analyses in pensioners revealed a tendency towards SES differences for the outcomes: other vascular diseases and number of comorbidities, but even here no clear gradients were observed. Moreover, temporal difference between the three income groups was very minimal (1%) and has no substantial meaning, which challenges potential conclusions on the possible existence of SES inequalities for this subgroup (pensioners). The results however highlight a slight potential towards socioeconomic inequalities in the development of T2D comorbidities in the population subgroup of pensioners, which needs to be further investigated at later time points. Moreover, the literature is short of studies on SES inequalities in the temporal change in T2D comorbidities, and this is, to our knowledge, is one of the first studies from Germany with this respect. Different countries have different socioeconomic constellations and health care systems, making it essential to examine time trends of potential SES disparities in the development of T2D comorbidities in order to address this research gap.

### Susceptible subgroups

The analyses showed that nonworking spouses with T2D are at a higher risk for comorbidities compared to working individuals, despite having similar time trends. Nonworking spouses of both genders had almost double the probability of having severe CVD comorbidities, a clearly higher probability for other comorbidities, and overall a higher number of comorbidities. Previous research using the same database indicated a higher prevalence of T2D in nonworking spouses compared to working individuals^8^. The association between employment and morbidity in individuals with T2D can follow different directions. One can argue that morbidity, or in this context having T2D complications, would lead to unemployment due to health impairment. While this can be true, the higher morbidity level in nonworking spouses could also be explained by the role accumulation theory. This suggests that having several social roles is associated with better health and health-promoting behaviors due to their effect on how people structure their daily activities. Having several social roles can be associated with the feeling of accomplishment that is socially valued, which also has a positive influence on self-esteem and well-being^19,52,53^. In any case, the relatively higher morbidity level in nonworking spouses highlights the importance of focusing on this population subgroup in T2D management interventions.

### Strengths and limitations

This study was done on a large population of a statutory health insurance provider in the state of lower Saxony, Germany, which includes all coded diagnoses and undergone treatments, limiting selection bias associated with surveys. The socioeconomic structure of the AOKN differs from that of the German population, which yet should not affect the generalizability of the results since analyses in this study were stratified by SES. One limitation of the study is transferring SES information for nonworking spouses from their employed partners. Results for this population subgroup should thus be considered under the presumption of household homogamy. Moreover, other population subgroups such as unemployed individuals could not be considered since no SES information is available and transferring SES information from family members is not possible. Thus, further studies should also focus on unemployed individuals and those with precarious job situations. Moreover, the income of pensioners cannot be compared 1:1 with the income of the working population. However, material resources in terms of purchasing power do not differ^54,55^, and the reduced pension income applies to all income levels within this subgroup*.* One more limitation is that the analyses are based on individual income, while in many studies household income or adjusted household income had been used^56^. In health insurance data, only individual income is available which may be considered a limiting condition. While this argument is reasonable, using this measure may not lead to serious bias as in an earlier comparison of five types of income (including individual income) social gradients emerged irrespective of the type considered^57^. Additionally, the predicted probabilities for T2D comorbidities could be overestimated, since T2D is often diagnosed after the onset of comorbidities. A further limitation of the study is that changes in coding practices of T2D comorbidities, such as changes in financial incentives, may have biased the observed trend results. This could be due to changes in how the data was recorded and reported over time. In the same vein, changes in treatment guidelines may also have had an effect on the trend results that were not accounted for in the study. Nevertheless, as our previous study using the same population and time-periods indicated, the temporal change of the single comorbidities within each comorbidity group exhibited similar attitudes^7^. Thus, the temporal change observed for the comorbidity index variables in this study was not due to a change in the coding behavior of one specific comorbidity.

## Conclusion

While it is well established that socioeconomically disadvantaged people are more likely to be affected by T2D, socioeconomic disparities do not exist in the prevalence and the trends of T2D comorbidities in a German population of statutory insured individuals in the state of Lower Saxony. The predicted probabilities of most T2D comorbidities and the number of comorbidities increased significantly between 2005 and 2017 in the examined population and SES subgroups and in both genders, indicating that morbidity expansion applies to the population of T2D regardless of social factors. However, the population subgroup nonworking spouses is a susceptible subgroup with markedly higher rates of T2D comorbidities compared to employed individuals, and needs to be focused upon when planning and implementing diabetes management interventions.

## Supplementary Information

Supplementary Information.

## Data Availability

The data underlying this study belong to the Allgemeine Ortskrankenkasse Niedersachsen (AOKN-General Local Health Insurance of Lower Saxony). The data are not publically available due to protection of data privacy of the insured individuals by the AOKN. Interested researchers can send data access requests to Dr. Jona Stahmeyer at the AOKN using the following e-mail address: Jona.Stahmeyer@aok.nds.de. The authors did not have any special access privileges.

## Abbreviations

T2D: Type 2 diabetes
SES: Socioeconomic status
CVD: Cardiovascular diseases
p1; p2; p3: Period 1 (2005–2007); period 2 (2010–2012); period 3 (2015–2017)
AOK; AOKN: Allgemeine Ortskrankenkasse; Allgemeine Ortskrankenkasse Niedersachsen
ICD-10: International classification of diseases version 10
WIdO: Scientific institute of AOK
AGI: German average annual income
DMP: Disease management program
