# Supplementary material for: Socioeconomic inequalities in type 2 diabetes comorbidities in different population subgroups: trend analyses using German health insurance data
Source: Sci Rep. 2023 Jul 5;13:10855. doi: 10.1038/s41598-023-37951-y (PMC10322827; doi:10.1038/s41598-023-37951-y)
Supplement: Supplementary file 1 — Supplementary Information. [file 41598_2023_37951_MOESM1_ESM.docx]

**Socioeconomic Inequalities in Type 2 Diabetes Comorbidities in Different Population Subgroups: Trend Analyses Using German Health Insurance Data**

**Supplementary Files**

**Table S1.** ICD-10 codes and type of diagnoses of the single comorbidities.

| **Comorbidity** | **ICD-10-GM Codes** | **Type of Diagnosis** |
| --- | --- | --- |
| **Myocardial Infarct** | I21 | Primary inpatient |
| **Stroke** | I60-I64 | Primary inpatient |
| **Angina Pectoris** |  |  |
| *Stable Angina* | I20.1, I20.8, I20.9 | Primary and secondary inpatient & confirmed outpatient |
| *Unstable Angina* | I20.0 | Primary and secondary inpatient & confirmed outpatient |
| **Hypertension** | I10-I15 | Primary and secondary inpatient & confirmed outpatient |
| **Hyperlipidemia** | E78 | Primary and secondary inpatient & confirmed outpatient |
| **Cardiac Insufficiency** | I50 | Primary and secondary inpatient & confirmed outpatient |
| **Retinopathy*** | H36 | Confirmed outpatient |
| **Nephropathy** | N18, N19, N08.3 | Primary and secondary inpatient & confirmed outpatient |
| **Polyneuropathy**** | G63 | Confirmed outpatient & secondary inpatient |
|  | Epilepsy medications: N03AX16, N03AX12 or N03AF01 but with no epilepsy diagnosis: G40-G41  Depression medications: N06AA09, N06AX21 but with no depression diagnosis: F30-F39 |  |

*No inpatient diagnoses were present in the data

**No primary inpatient diagnoses were present in the data

Reference: (1)

**Table S2.** Reference income figures in Euros and their equivalents for the three income levels for the three time periods of the study.

|  |  | **AGI*** | **Adjusted† AGI** | **<60% AGI** | **60%-80% AGI** | **>80% AGI** |
| --- | --- | --- | --- | --- | --- | --- |
| **Period 1** | **2005** | 29.202 € | 25.406 € | < 15.243 € | 15.243 € - 20.325 € | > 20.325 € |
|  | **2006** | 29.494 € | 25.660 € | < 15.396 € | 15.396 € - 20.528 € | > 20.528 € |
|  | **2007** | 29.951 € | 26.342 € | < 15.805 € | 15.805 € - 21.074 € | > 21.074 € |
| **Period 2** | **2010** | 31.144 € | 27.609 € | < 16.565 € | 16.565 € - 22.087 € | > 22.087 € |
|  | **2011** | 32.100 € | 28.425 € | < 17.055 € | 17.055 € - 22.740 € | > 22.740 € |
|  | **2012** | 33.002 € | 29.273 € | < 17.564 € | 17.564 € - 23.418€ | > 23.418 € |
| **Period 3** | **2015** | 35.363 € | 31.526 € | < 18.916 € | 18.916 € - 25.221 € | > 25.221 € |
|  | **2016** | 36.187 € | 32.261 € | < 19.356 € | 19.356 € - 25.809 € | > 25.809 € |
|  | **2017** | 37.077 € | 33.054 € | < 19.832 € | 19.832 € - 26.443 € | > 26.443 € |
| *AGI is the average German annual income of the former Western federal states of Germany in terms of salary of working individuals as reported by the Federal Statistical Office of Germany. Reference: (2, 3) †AGI adjusted for unemployment insurance and pension insurance contributions | | | | | | |

**Table S3.** Socioeconomic trends in the comorbidities group: **Less severe CVD (hypertension, hyperlipidemia, cardiac insufficiency)** illustrated by 2-sided interactions between time and SES with the corresponding reference groups: period 1 (for the variable time period) * low income (for the variable income), ≤ 9 years of schooling (for the variable school education) and manuals (for the variable occupation). Stratified by gender and population subgroup. Estimated by means of logistic regression, adjusting for age and duration of observation and correcting for within cluster variation. ***Income*** Low: <60% AGI, Middle: 60%-80% AGI, Higher: >80% AGI.

|  | **Men** | | | | | | **Women** | | | | | | |
| --- | --- | --- | --- | --- | --- | --- | --- | --- | --- | --- | --- | --- | --- |
|  | **Working individuals** | | **Nonworking Spouses** | | **Pensioners** | | **Working individuals** | | **Nonworking Spouses** | | **Pensioners** | |  |
|  | **OR** | **CI** | **OR** | **CI** | **OR** | **CI** | **OR** | **CI** | **OR** | **CI** | **OR** | **CI** |  |
| ***Income*** |  |  |  |  |  |  |  |  |  |  |  |  |  |
| Middle income * Period 2 | 1 | 0,81 - 1,12 | 0,9 | 0,53 - 1,42 | 1,02 | 0,96 - 1,09 | 1 | 0,87 - 1,19 | 0,94 | 0,78 - 1,13 | 1,02 | 0,93 - 1,12 |  |
| Higher income * Period 2 | 0,9 | 0,83 - 1,06 | 0,7 | 0,53 - 0,9 | 0,92 | 0,85 - 1,01 | 1,1 | 0,99 - 1,28 | **0,78*** | 0,69 - 0,89 | 0,91 | 0,82 - 1,02 |  |
| Middle income * Period 3 | 1,1 | 0,9 - 1,24 | 1 | 0,58 - 1,7 | 1,05 | 0,96 - 1,15 | 1,1 | 0,92 - 1,27 | 0,89 | 0,72 - 1,11 | 1,1 | 0,98 - 1,24 |  |
| Higher income * Period 3 | 1 | 0,85 - 1,09 | 0,9 | 0,68 - 1,29 | 0,91 | 0,82 - 1,02 | 1 | 0,9 - 1,17 | 0,84 | 0,72 - 0,99 | 0,9 | 0,79 - 1,02 |  |
| ***Education*** |  |  |  |  |  |  |  |  |  |  |  |  |  |
| 10 years schooling * period 2 | 1 | 0,91 - 1,17 | 1,1 | 0,63 - 1,76 | - | - | 1 | 0,87 - 1,24 | 1,03 | 0,82 - 1,29 | - | - |  |
| 12-13 years schooling * period 2 | 1 | 0,77 - 1,21 | 1 | 0,45 - 2,26 | - | - | 1,2 | 0,89 - 1,68 | 0,6 | 0,38 - 0,95 | - | - |  |
| 10 years schooling * period 3 | 1 | 0,9 - 1,17 | 0,5 | 0,29 - 0,93 | - | - | 1,1 | 0,9 - 1,31 | 0,96 | 0,74 - 1,23 | - | - |  |
| 12-13 years schooling * period 3 | 1 | 0,78 - 1,27 | 0,6 | 0,24 - 1,41 | - | - | 1 | 0,75 - 1,43 | 0,64 | 0,39 - 1,06 | - | - |  |
| ***Occupation*** |  |  |  |  |  |  |  |  |  |  |  |  |  |
| Specialists * Period 2 | 1 | 0,92 - 1,2 | 1,1 | 0,7 - 1,8 | - | - | 1 | 0,84 - 1,11 | 1,1 | 0,79 - 1,43 | - | - |  |
| Highly qualified * Period 2 | 1 | 0,74 - 1,23 | 0,4 | 0,15 - 1,37 | - | - | 1,4 | 1 - 2,07 | 0,7 | 0,39 - 1,2 | - | - |  |
| Specialists * Period 3 | 1,1 | 0,96 - 1,28 | 1 | 0,57 - 1,74 | - | - | 1 | 0,86 - 1,14 | 1 | 0,76 - 1,42 | - | - |  |
| Highly qualified * Period 3 | 1,1 | 0,87 - 1,49 | 0,7 | 0,03 - 0,13 | - | - | 1,3 | 0,9 - 1,92 | 0,6 | 0,36 - 1,17 | - | - |  |
| *p<0,001 |  |  |  |  |  |  |  |  |  |  |  |  |  |
| **Table S4.** Socioeconomic trends in the comorbidities group: **More severe CVD (myocardial infarction, stroke, angina pectoris)** illustrated by 2-sided interactions between time and SES with the corresponding reference groups: period 1 (for the variable time period) * low income (for the variable income), ≤ 9 years of schooling (for the variable school education) and manuals (for the variable occupation). Stratified by gender and population subgroup. Estimated by means of logistic regression, adjusting for age and duration of observation and correcting for within cluster variation. ***Income*** Low: <60% AGI, Middle: 60%-80% AGI, Higher: >80% AGI. | | | | | | | | | | | | |  |
|  | **Men** | | | | | | **Women** | | | | | | |
|  | **Working individuals** | | **Nonworking Spouses** | | **Pensioners** | | **Working individuals** | | **Nonworking Spouses** | | **Pensioners** | |  |
|  | **OR** | **CI** | **OR** | **CI** | **OR** | **CI** | **OR** | **CI** | **OR** | **CI** | **OR** | **CI** |  |
| ***Income*** |  |  |  |  |  |  |  |  |  |  |  |  |  |
| Middle income * Period 2 | 1 | 0,75 - 1,31 | 1,5 | 0,78 - 2,67 | 1 | 0,94 - 1,06 | 1,2 | 0,77 - 1,75 | 0,87 | 0,67 - 1,15 | 0,94 | 0,87 - 1,01 |  |
| Higher income * Period 2 | 1,2 | 0,99 - 1,46 | 1,1 | 0,81 - 1,52 | 0,96 | 0,89 - 1,05 | 1,2 | 0,82 - 1,61 | 1 | 0,82 - 1,23 | **0,79*** | 0,72 - 0,88 |  |
| Middle income * Period 3 | 1,1 | 0,84 - 1,42 | 1,5 | 0,84 - 2,82 | 0,95 | 0,89 - 1,01 | 1,2 | 0,82 - 1,83 | 1,15 | 0,85 - 1,55 | 0,95 | 0,88 - 1,03 |  |
| Higher income * Period 3 | 1,2 | 0,99 - 1,46 | 1,2 | 0,86 - 1,65 | 0,96 | 0,88 - 1,06 | 1,4 | 0,97 - 1,88 | 1,02 | 0,81 - 1,3 | 0,9 | 0,81 - 1,01 |  |
| ***Education*** |  |  |  |  |  |  |  |  |  |  |  |  |  |
| 10 years schooling * period 2 | 1,3 | 0,93 - 1,69 | 0,8 | 0,36 - 1,57 | - | - | 1,2 | 0,69 - 2,25 | 1,21 | 0,57 - 2,57 | - | - |  |
| 12-13 years schooling * period 2 | 1 | 0,56 - 1,70 | 1,3 | 0,42 - 4,2 | - | - | 0,8 | 0,3 - 1,96 | 0,45 | 0,15 - 1,31 | - | - |  |
| 10 years schooling * period 3 | 1,1 | 0,82 - 1,5 | 0,8 | 0,37 - 1,56 | - | - | 0,8 | 0,43 - 1,43 | 1,57 | 0,75 - 3,3 | - | - |  |
| 12-13 years schooling * period 3 | 0,8 | 0,48 - 1,43 | 1,3 | 0,4 - 4,39 | - | - | 0,5 | 0,19 - 1,18 | 0,18 | 0,05 - 0,64 | - | - |  |
| ***Occupation*** |  |  |  |  |  |  |  |  |  |  |  |  |  |
| Specialists * Period 2 | 1,1 | 0,85 - 1,46 | 0,9 | 0,47 - 1,61 | - | - | 0,9 | 0,6 - 1,24 | 1,2 | 0,48 - 2,75 | - | - |  |
| Highly qualified * Period 2 | 1,1 | 0,64 - 1,9 | 1,3 | 0,14 - 12.5 | - | - | 1,6 | 0,35 - 7,44 | 1,1 | 0,23 - 5,33 | - | - |  |
| Specialists * Period 3 | 1 | 0,8 - 1,37 | 0,7 | 0,37 - 1,42 | - | - | 0,7 | 0,47 - 1 | 1 | 0,41 - 2,4 | - | - |  |
| Highly qualified * Period 3 | 0,9 | 0,5 - 1,47 | 0,7 | 0,06 - 7,58 | - | - | 2,5 | 0,58 - 10,9 | 0,7 | 0,14 - 3,56 | - | - |  |
| *p<0,001 |  |  |  |  |  |  |  |  |  |  |  |  |  |
|  |  |  |  |  |  |  |  |  |  |  |  |  |  |
| **Table S5.** Socioeconomic trends in the comorbidities group: **Other vascular diseases (nephropathy, polyneuropathy, retinopathy)** illustrated by 2-sided interactions between time and SES with the corresponding reference groups: period 1 (for the variable time period) * low income (for the variable income), ≤ 9 years of schooling (for the variable school education) and manuals (for the variable occupation). Stratified by gender and population subgroup. Estimated by means of logistic regression, adjusting for age and duration of observation and correcting for within cluster variation. ***Income*** Low: <60% AGI, Middle: 60%-80% AGI, Higher: >80% AGI. | | | | | | | | | | | | |  |
|  | **Men** | | | | | | **Women** | | | | | | |
|  | **Working individuals** | | **Nonworking Spouses** | | **Pensioners** | | **Working individuals** | | **Nonworking Spouses** | | **Pensioners** | |  |
|  | **OR** | **CI** | **OR** | **CI** | **OR** | **CI** | **OR** | **CI** | **OR** | **CI** | **OR** | **CI** |  |
| ***Income*** |  |  |  |  |  |  |  |  |  |  |  |  |  |
| Middle income * Period 2 | 1 | 0,81 - 1,23 | 1,4 | 0,88 - 2,3 | 1,02 | 0,98 - 1,07 | 1 | 0,83 - 1,26 | 1,03 | 0,88 - 1,2 | 0,98 | 0,93 - 1,02 |  |
| Higher income * Period 2 | 1,1 | 0,95 - 1,26 | 1 | 0,78 - 1,27 | 0,91 | 0,86 - 0,97 | 1,1 | 0,96 - 1,35 | 0,97 | 0,86 - 1,09 | **0,83*** | 0,78 - 0,89 |  |
| Middle income * Period 3 | 1 | 0,85 - 1,26 | 1,3 | 0,78 - 2,05 | 1 | 0,96 - 1,05 | 1,1 | 0,86 - 1,28 | 1,01 | 0,86 - 1,2 | 1,01 | 0,96 - 1,07 |  |
| Higher income * Period 3 | 1,1 | 0,93 - 1,22 | 1 | 0,74 - 1,23 | **0,86*** | 0,81 - 0,92 | 1,2 | 0,98 - 1,36 | 0,96 | 0,85 - 1,09 | **0,85*** | 0,79 - 0,92 |  |
| ***Education*** |  |  |  |  |  |  |  |  |  |  |  |  |  |
| 10 years schooling * period 2 | 0,8 | 0,68 - 0,99 | 0,8 | 0,49 - 1,44 | - | - | 1 | 0,73 - 1,3 | 0,83 | 0,59 - 1,16 | - | - |  |
| 12-13 years schooling * period 2 | 1 | 0,72 - 1,5 | 0,9 | 0,44 - 1,93 | - | - | 0,9 | 0,52 - 1,47 | 0,71 | 0,34 - 1,5 | - | - |  |
| 10 years schooling * period 3 | 0,8 | 0,66 - 0,95 | 0,8 | 0,45 - 1,39 | - | - | 1 | 0,73 - 1,29 | 0,92 | 0,66 - 1,28 | - | - |  |
| 12-13 years schooling * period 3 | 1,1 | 0,74 - 1,57 | 0,6 | 0,27 - 1,27 | - | - | 0,7 | 0,43 - 1,2 | 0,68 | 0,34 - 1,39 | - | - |  |
| ***Occupation*** |  |  |  |  |  |  |  |  |  |  |  |  |  |
| Specialists * Period 2 | 0,9 | 0,72 - 1 | 1,2 | 0,74 - 2,05 | - | - | 0,9 | 0,78 - 1,13 | 0,7 | 0,47 - 0,95 | - | - |  |
| Highly qualified * Period 2 | 1 | 0,68 - 1,4 | 0,7 | 0,23 - 2 | - | - | 1,6 | 0,83 - 3,07 | 1,9 | 0,73 - 5,06 | - | - |  |
| Specialists * Period 3 | 0,9 | 0,7 - 1 | 1,1 | 0,65 - 1,91 | - | - | 0,9 | 0,78 - 1,11 | 0,8 | 0,59 - 1,18 | - | - |  |
| Highly qualified * Period 3 | 1 | 0,68 - 1,35 | 0,8 | 0,25 - 2,53 | - | - | 1,6 | 0,86 - 3,15 | 2,2 | 0,84 - 5,8 | - | - |  |
| *p<0,001 |  |  |  |  |  |  |  |  |  |  |  |  |  |
|  |  |  |  |  |  |  |  |  |  |  |  |  |  |
|  |  |  |  |  |  |  |  |  |  |  |  |  |  |
| **Table S6.** Socioeconomic trends in the outcome: **Number of comorbidities (with the 4 categories: 0, 1, 2 & >2 comorbidities)** illustrated by 2-sided interactions between time and SES with the corresponding reference groups: period 1 (for the variable time period) * low income (for the variable income), ≤ 9 years of schooling (for the variable school education) and manuals (for the variable occupation). Stratified by gender and population subgroup. Estimated by means of ordinal regression, adjusting for age and duration of observation and correcting for within cluster variation. ***Income*** Low: <60% AGI, Middle: 60%-80% AGI, Higher: >80% AGI. | | | | | | | | | | | | |  |
|  | **Men** | | | | | | **Women** | | | | | | |
|  | **Working individuals** | | **Nonworking Spouses** | | **Pensioners** | | **Working individuals** | | **Nonworking Spouses** | | **Pensioners** | |  |
|  | **OR** | **CI** | **OR** | **CI** | **OR** | **CI** | **OR** | **CI** | **OR** | **CI** | **OR** | **CI** |  |
| ***Income*** |  |  |  |  |  |  |  |  |  |  |  |  |  |
| Middle income * Period 2 | 1 | 0,88 - 1,15 | 1,2 | 0,85 - 1,63 | 0,99 | 0,95 - 1,03 | 1 | 0,89 - 1,15 | 0,97 | 0,86 - 1,08 | 0,96 | 0,92 - 1 |  |
| Higher income * Period 2 | 1 | 0,93 - 1,13 | 0,9 | 0,72 - 1,05 | **0,89*** | 0,85 - 0,94 | 1,1 | 0,97 - 1,19 | 0,89 | 0,81 - 0,97 | **0,88*** | 0,83 - 0,94 |  |
| Middle income * Period 3 | 1 | 0,89 - 1,16 | 1,1 | 0,77 - 1,53 | 1,02 | 0,98 - 1,07 | 1,1 | 0,92 - 1,19 | 0,96 | 0,84 - 1,1 | 0,97 | 0,93 - 1,02 |  |
| Higher income * Period 3 | 1 | 0,89 - 1,09 | 1 | 0,82 - 1,23 | **0,86*** | 0,8 - 0,91 | 1 | 0,93 - 1,14 | 0,95 | 0,86 - 1,05 | **0,86*** | 0,81 - 0,92 |  |
| ***Education*** |  |  |  |  |  |  |  |  |  |  |  |  |  |
| 10 years schooling * period 2 | 1 | 0,93 - 1,14 | 1 | 0,65 - 1,38 | - | - | 1 | 0,84 - 1,13 | 0,99 | 0,82 - 1,19 | - | - |  |
| 12-13 years schooling * period 2 | 1 | 0,8 - 1,17 | 1,2 | 0,7 - 2,15 | - | - | 1,1 | 0,81 - 1,46 | 0,74 | 0,49 - 1,13 | - | - |  |
| 10 years schooling * period 3 | 1 | 0,89 - 1,1 | 0,9 | 0,57 - 1,28 | - | - | 1 | 0,83 - 1,13 | 0,94 | 0,77 - 1,15 | - | - |  |
| 12-13 years schooling * period 3 | 1 | 0,83 - 1,24 | 0,9 | 0,49 - 1,6 | - | - | 0,9 | 0,65 - 1,18 | 0,65 | 0,43 - 1 | - | - |  |
| ***Occupation*** |  |  |  |  |  |  |  |  |  |  |  |  |  |
| Specialists * Period 2 | 1 | 0,89 - 1,1 | 1 | 0,7 - 1,38 | - | - | 0,9 | 0,83 - 1,04 | 0,9 | 0,67 - 1,11 | - | - |  |
| Highly qualified * Period 2 | 1 | 0,78 - 1,17 | 0,7 | 0,36 - 1,42 | - | - | 1,2 | 0,86 - 1,66 | 0,8 | 0,55 - 1,28 | - | - |  |
| Specialists * Period 3 | 1 | 0,91 - 1,14 | 0,8 | 0,58 - 1,23 | - | - | 0,9 | 0,81 - 1 | 1 | 0,73 - 1,25 | - | - |  |
| Highly qualified * Period 3 | 1 | 0,81 - 1,21 | 1 | 0,48 - 2,03 | - | - | 1,2 | 0,82 - 1,62 | 0,8 | 0,5 - 1,22 | - | - |  |
| *p<0,001 |  |  |  |  |  |  |  |  |  |  |  |  |  |

**Table S7.** Prevalence/odds ratios and confidence intervals on the effect of time period on the 3 comorbidity-index variables and the number of comorbidities, stratified by gender, population subgroup and **years of school education**. Estimated by means of logistic regression and ordinal regression, adjusting for within cluster variation. Adjusted for age and insurance duration. ***Time periods*** p1 (reference): 2005-2007, p2: 2010-2012, p3:2015-2017. ***Comorbidities*** Less severe CVD: Hypertension, Hyperlipidemia, Cardiac insufficiency; More severe CVD: Myocardial infarction, Stroke, Angina Pectoris; Other vascular diseases: Nephropathy, Neuropathy, Retinopathy.

|  |  | **Less Severe CVD CMs** | | | | **More Severe CVD CMs** | | | | **Other Vascular Diseases** | | | | **Number of Comorbidities** | | | |
| --- | --- | --- | --- | --- | --- | --- | --- | --- | --- | --- | --- | --- | --- | --- | --- | --- | --- |
|  | **n** | **p2** | | **p3** | | **p2** | | **p3** | | **p2** | | **p3** | | **p2** | | **p3** | |
| ***Men*** |  | **PR** | **95% CI** | **PR** | **95% CI** | **PR** | **95% CI** | **PR** | **95% CI** | **PR** | **95% CI** | **PR** | **95% CI** | **OR** | **95% CI** | **OR** | **95% CI** |
| **Employed** |  |  |  |  |  |  |  |  |  |  |  |  |  |  |  |  |  |
| ≤ 9 years | 29168 | 1,08 | 1,06 - 1,10 | 1,17 | 1,14 - 1,19 | 1,19 | 1,01 - 1,37 | 1,25 | 1,06 - 1,45 | 1,58 | 1,43 - 1,73 | 2,14 | 1,94 - 2,35 | 1,43 | 1,36 - 1,51 | 2,25 | 2,12 - 2,4 |
| 10 years | 14137 | 1,1 | 1,06 - 1,13 | 1,18 | 1,14 - 1,21 | 1,41 | 1,06 - 1,76 | 1,31 | 0,98 - 1,65 | 1,31 | 1,13 - 1,48 | 1,71 | 1,48 - 1,95 | 1,48 | 1,36 - 1,61 | 2,23 | 2,03 - 2,44 |
| 12-13 years | 3184 | 1,07 | 1,01 - 1,14 | 1,17 | 1,09 - 1,24 | 1,13 | 0,56 - 1,70 | 1,02 | 0,52 - 1,52 | 1,6 | 1,09 - 2,1 | 2,18 | 1,47 - 2,89 | 1,35 | 1,12 - 1,62 | 2,18 | 1,79 - 2,66 |
| **Non-working Spouses** |  |  |  |  |  |  |  |  |  |  |  |  |  |  |  |  |  |
| ≤ 9 years | 1275 | 1,06 | 1 - 1,13 | 1,16 | 1,08 - 1,24 | 0,97 | 0,64 - 1,30 | 1,04 | 0,65 - 1,44 | 1,46 | 1,06 - 1,87 | 1,96 | 1,37 - 2,54 | 1,39 | 1,09 - 1,77 | 2,22 | 1,69 - 2,91 |
| 10 years | 825 | 1,05 | 0,98 - 1,12 | 1,04 | 0,96 - 1,12 | 0,78 | 0,32 - 1,24 | 0,87 | 0,37 - 1,36 | 1,28 | 0,85 - 1,71 | 1,62 | 1,04 - 2,2 | 1,34 | 1 - 1,81 | 1,92 | 1,35 - 2,73 |
| 12-13 years | 269 | 1,06 | 0,92 - 1,2 | 1,08 | 0,93 - 1,23 | 1,26 | 0 - 2,53 | 1,38 | 0 - 2,9 | 1,31 | 0,65 - 1,96 | 1,21 | 0,53 - 1,89 | 1,72 | 1,04 - 2,86 | 1,94 | 1,10 - 3,41 |
| ***Women*** |  |  |  |  |  |  |  |  |  |  |  |  |  |  |  |  |  |
| **Employed** |  |  |  |  |  |  |  |  |  |  |  |  |  |  |  |  |  |
| ≤ 9 years | 11126 | 1,08 | 1,04 - 1,12 | 1,16 | 1,12 - 1,21 | 1,11 | 0,74 - 1,49 | 1,2 | 0,78 - 1,62 | 1,58 | 1,34 - 1,83 | 2,12 | 1,79 - 2,44 | 1,39 | 1,27 - 1,53 | 2,21 | 2 - 2,45 |
| 10 years | 9398 | 1,1 | 1,05 - 1,14 | 1,2 | 1,14 - 1,25 | 1,3 | 0,69 - 1,91 | 0,88 | 0,46 - 1,30 | 1,54 | 1,21 - 1,86 | 2,04 | 1,61 - 2,48 | 1,36 | 1,20 - 1,54 | 2,14 | 1,89 - 2,43 |
| 12-13 years | 2295 | 1,16 | 1,04 - 1,28 | 1,21 | 1,08 - 1,34 | 0,78 | 0,13 - 1,43 | 0,51 | 0,1 - 0,92 | 1,39 | 0,77 - 2 | 1,6 | 0,9 - 2,3 | 1,47 | 1,12 - 1,94 | 1,86 | 1,41 - 2,45 |
| **Non-working Spouses** |  |  |  |  |  |  |  |  |  |  |  |  |  |  |  |  |  |
| ≤ 9 years | 7793 | 1,04 | 1,02 - 1,06 | 1,08 | 1,06 - 1,11 | 1,24 | 0,81 - 1,66 | 1,21 | 0,78 - 1,64 | 1,48 | 1,26 - 1,7 | 1,81 | 1,54 - 2,09 | 1,33 | 1,21 - 1,48 | 2,04 | 1,83 - 2,29 |
| 10 years | 3231 | 1,04 | 1,01 - 1,07 | 1,07 | 1,03 - 1,1 | 1,57 | 0,54 - 2,6 | 2,03 | 0,65 - 3,41 | 1,27 | 0,97 - 1,57 | 1,68 | 1,29 - 2,08 | 1,31 | 1,10 - 1,55 | 1,89 | 1,57 - 2,27 |
| 12-13 years | 592 | 0,96 | 0,9 - 1,02 | 1,01 | 0,94 - 1,07 | 0,61 | 0 - 1,27 | 0,21 | 0 - 0,52 | 1,11 | 0,50 - 1,72 | 1,31 | 0,63 - 1,98 | 0,94 | 0,63 - 1,39 | 1,21 | 0,8 - 1,81 |

**Table S8.** Prevalence/odds ratios and confidence intervals on the effect of time period on the 3 comorbidity-index variables less severe CVD, more severe CVD and other vascular diseases and the number of comorbidities, stratified by gender, population subgroup and **occupation group**. Estimated by means of logistic regression and ordinal regression, adjusting for within cluster variation. Adjusted for age and insurance duration. ***Time periods*** p1 (reference): 2005-2007, p2: 2010-2012, p3:2015-2017. ***Comorbidities*** Less severe CVD: Hypertension, Hyperlipidemia, Cardiac insufficiency; More severe CVD: Myocardial infarction, Stroke, Angina Pectoris; Other vascular diseases: Nephropathy, Neuropathy, Retinopathy.

|  |  | **Less Severe CVD CMs** | | | | **More Severe CVD CMs** | | | | **Other Vascular Diseases** | | | | **Number of Comorbidities** | | | |
| --- | --- | --- | --- | --- | --- | --- | --- | --- | --- | --- | --- | --- | --- | --- | --- | --- | --- |
|  | **n** | **p2** | | **p3** | | **p2** | | **p3** | | **p2** | | **p3** | | **p2** | | **p3** | |
| ***Men*** |  | **PR** | **95% CI** | **PR** | **95% CI** | **PR** | **95% CI** | **PR** | **95% CI** | **PR** | **95% CI** | **PR** | **95% CI** | **OR** | **95% CI** | **OR** | **95% CI** |
| **Employed** |  |  |  |  |  |  |  |  |  |  |  |  |  |  |  |  |  |
| Manuals | 64208 | 1,1 | 1,08 - 1,1 | 1,2 | 1,16 - 1,19 | 1 | 0,93 - 1,1 | 1 | 0,92 - 1,1 | 1,4 | 1,31 - 1,45 | 1,8 | 1,71 - 1,9 | 1,3 | 1,35 - 1,44 | 2,1 | 2,05 - 2,2 |
| Specialists | 8889 | 1,1 | 1,07 - 1,14 | 1,2 | 1,16 - 1,24 | 1,1 | 0,84 - 1,38 | 1,1 | 0,79 - 1,31 | 1,2 | 1,04 - 1,34 | 1,5 | 1,35 - 1,75 | 1,4 | 1,24 - 1,52 | 2,1 | 1,93 - 2,39 |
| Highly Qualified | 3371 | 1,1 | 1 - 1,16 | 1,2 | 1,12 - 1,3 | 1,1 | 0,52 - 1,62 | 0,9 | 0,42 - 1,29 | 1,3 | 0,92 - 1,73 | 1,7 | 1,21 - 2,25 | 1,4 | 1,1 - 1,66 | 2,2 | 1,76 - 2,67 |
| **Non-working Spouses** |  |  |  |  |  |  |  |  |  |  |  |  |  |  |  |  |  |
| Manuals | 3084 | 1,1 | 1,02 - 1,12 | 1,2 | 1,1 - 1,2 | 1,1 | 0,82 - 1,31 | 1,1 | 0,8 - 1,34 | 1,6 | 1,29 - 1,8 | 2,1 | 1,72 - 2,44 | 1,5 | 1,25 - 1,67 | 2,8 | 1,92 - 2,71 |
| Specialists | 716 | 1,1 | 0,99 - 1,17 | 1,1 | 1,02 - 1,22 | 1 | 0,47 - 1,43 | 0,8 | 0,34 - 1,23 | 1,9 | 1,16 - 2,61 | 2,3 | 1,39 - 3,27 | 1,4 | 1,05 - 1,97 | 1,9 | 1,36 - 2,74 |
| Highly Qualified | 159 | 0,9 | 0,7 - 1,14 | 1,1 | 0,84 - 1,31 | 1,6 | 0 - 5,1 | 0,8 | 0 - 2,57 | 1,1 | 0,1 - 2,09 | 1,8 | 0,11 - 3,43 | 1,1 | 0,5 - 2,26 | 2,6 | 1,14 - 5,71 |
| ***Women*** |  |  |  |  |  |  |  |  |  |  |  |  |  |  |  |  |  |
| **Employed** |  |  |  |  |  |  |  |  |  |  |  |  |  |  |  |  |  |
| Manuals | 27641 | 1,1 | 1,06 - 1,1 | 1,2 | 1,14 - 1,19 | 0,9 | 0,77 - 1,08 | 0,8 | 0,68 - 0,95 | 1,3 | 1,21 - 1,41 | 1,7 | 1,55 - 1,8 | 1,3 | 1,24 - 1,38 | 2 | 1,87 - 2,09 |
| Specialists | 10378 | 1,1 | 1,03 - 1,11 | 1,2 | 1,12 - 1,2 | 0,8 | 0,55 - 1,04 | 0,6 | 0,37 - 0,72 | 1,2 | 1,07 - 1,42 | 1,6 | 1,36 - 1,79 | 1,2 | 1,1 - 1,33 | 1,8 | 1,61 - 1,95 |
| Highly Qualified | 1576 | 1,2 | 1,06 - 1,38 | 1,3 | 1,11 - 1,46 | 1,5 | 0 - 3,76 | 2,1 | 0 - 5,03 | 2 | 0,81 - 3,19 | 2,6 | 1,07 - 4,18 | 1,6 | 1,14 - 2,19 | 2,3 | 1,65 - 3,26 |
| **Non-working Spouses** |  |  |  |  |  |  |  |  |  |  |  |  |  |  |  |  |  |
| Manuals | 17293 | 1,1 | 1,05 - 1,1 | 1,1 | 1,11 - 1,16 | 1 | 0,85 - 1,23 | 1 | 0,8 - 1,18 | 1,3 | 1,19 - 1,4 | 1,6 | 1,45 - 1,71 | 1,3 | 1,23 - 1,4 | 1,9 | 1,76 - 2,02 |
| Specialists | 1614 | 1,1 | 1,01 - 1,2 | 1,2 | 1,06 - 1,27 | 1,2 | 0,22 - 2,18 | 1 | 0,16 - 1,89 | 0,9 | 0,66 - 1,18 | 1,4 | 0,99 - 1,71 | 1,1 | 0,9 - 1,44 | 1,8 | 1,38 - 2,28 |
| Highly Qualified | 586 | 1 | 0,83 - 1,08 | 1 | 0,87 - 1,13 | 1,1 | 0 - 2,86 | 0,7 | 0 - 1,81 | 2,4 | 0,27 - 4,44 | 3,2 | 0,36 - 6,09 | 1,1 | 0,71 - 1,65 | 1,4 | 0,92 - 2,26 |

**Figure S1.** Predicted probabilities of comorbidity groups over the three time periods for **men**, stratified by **school** **education** (displayed as years of schooling) and population subgroup. ***Time periods*** p1: 2005-2007, p2: 2010-2012, p3:2015-2017. ***Comorbidities*** Less severe CVD: Hypertension, Hyperlipidemia, Cardiac insufficiency; More severe CVD: Myocardial infarction, Stroke, Angina Pectoris; Other vascular diseases: Nephropathy, Neuropathy, Retinopathy.


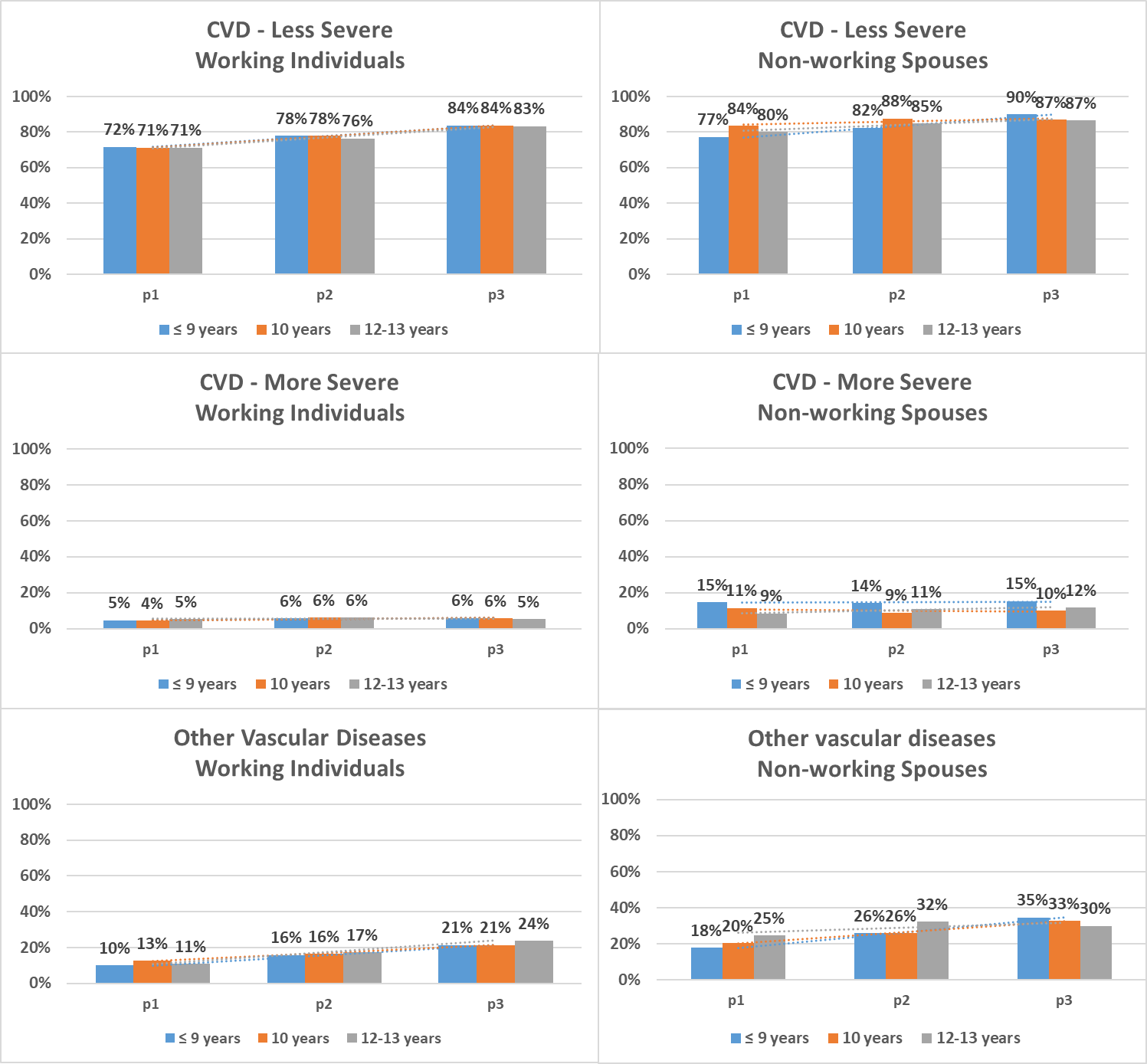


**Figure S2.** Predicted probabilities of comorbidity groups over the three time periods for **women**, stratified by **school** **education** (displayed as years of schooling) and population subgroup. ***Time periods*** p1: 2005-2007, p2: 2010-2012, p3:2015-2017. ***Comorbidities*** Less severe CVD: Hypertension, Hyperlipidemia, Cardiac insufficiency; More severe CVD: Myocardial infarction, Stroke, Angina Pectoris; Other vascular diseases: Nephropathy, Neuropathy, Retinopathy.
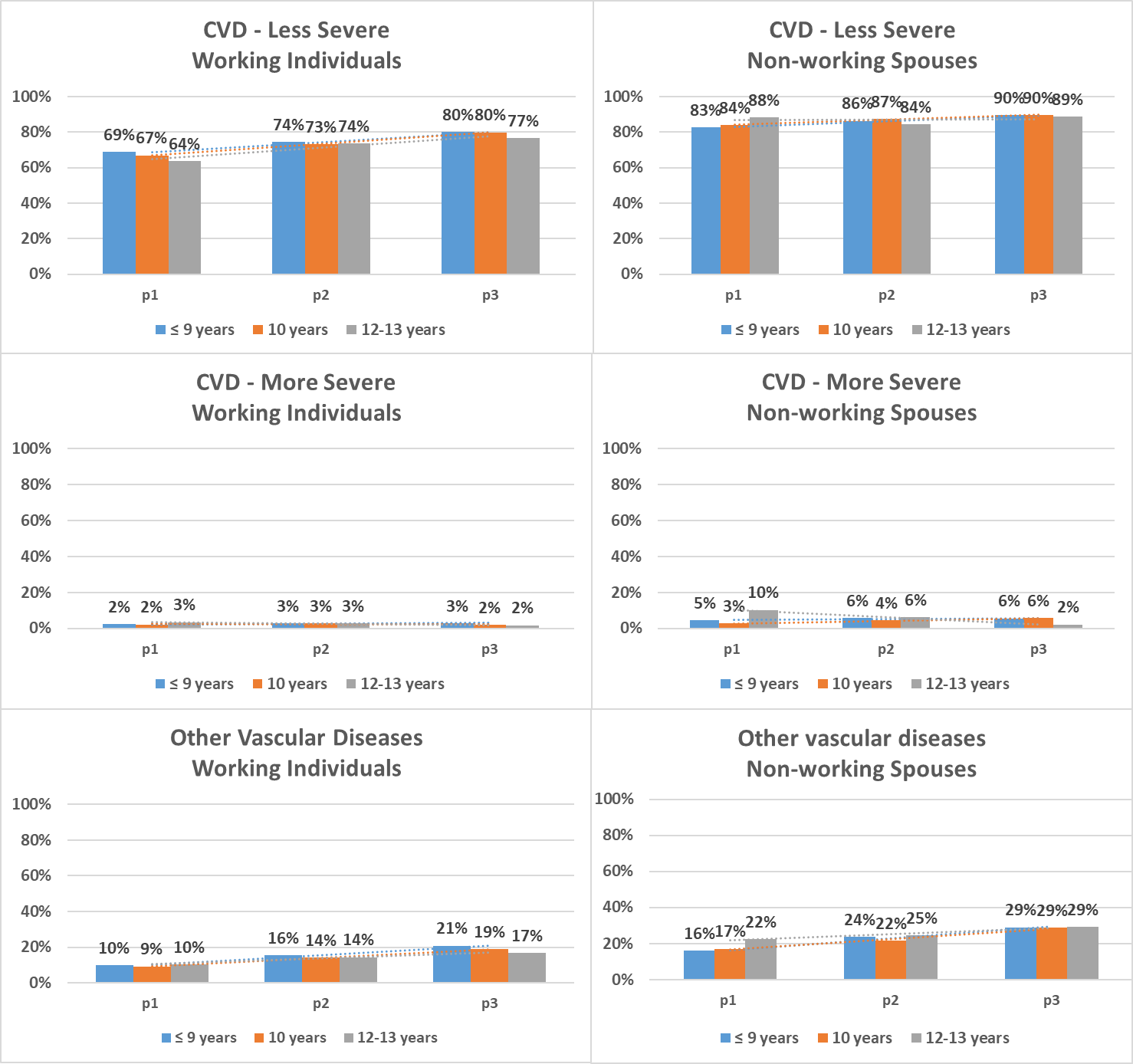


**Figure S3.** Predicted probabilities of the number of comorbidities over the three time periods for **men**, stratified by **school** **education** (displayed as years of schooling) and population subgroup. ***Time periods*** p1: 2005-2007, p2: 2010-2012, p3:2015-2017.


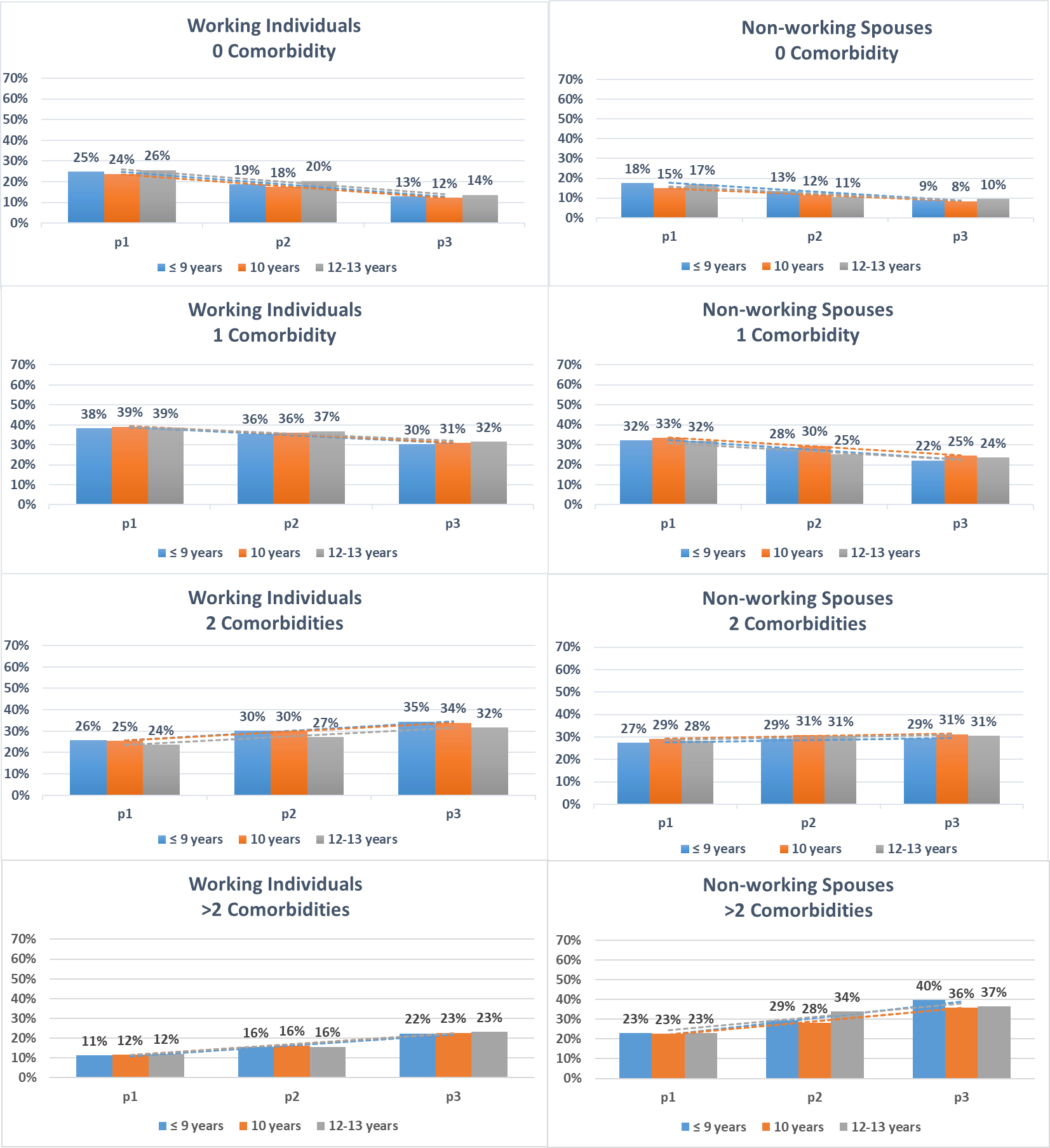


**Figure S4.** Predicted probabilities of the number of comorbidities over the three time periods for **women**, stratified by **school** **education** (displayed as years of schooling) and population subgroup. ***Time periods*** p1: 2005-2007, p2: 2010-2012, p3:2015-2017.


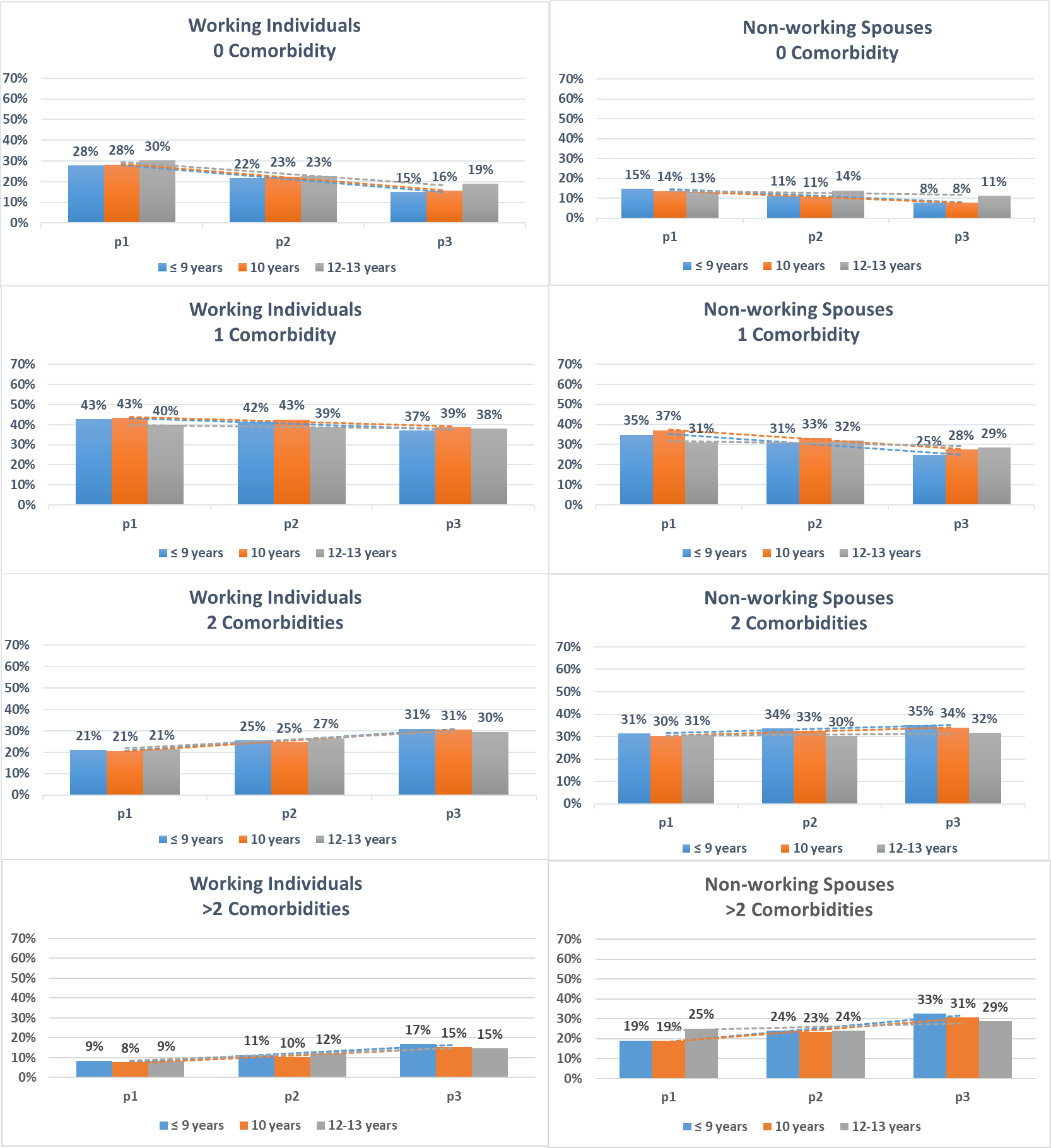


**Figure S5.** Predicted probabilities of comorbidity groups over the three time periods for **men**, stratified by **occupation** and population subgroup. ***Time periods*** p1: 2005-2007, p2: 2010-2012, p3:2015-2017. ***Comorbidities*** Less severe CVD: Hypertension, Hyperlipidemia, Cardiac insufficiency; More severe CVD: Myocardial infarction, Stroke, Angina Pectoris; Other vascular diseases: Nephropathy, Neuropathy, Retinopathy.

**
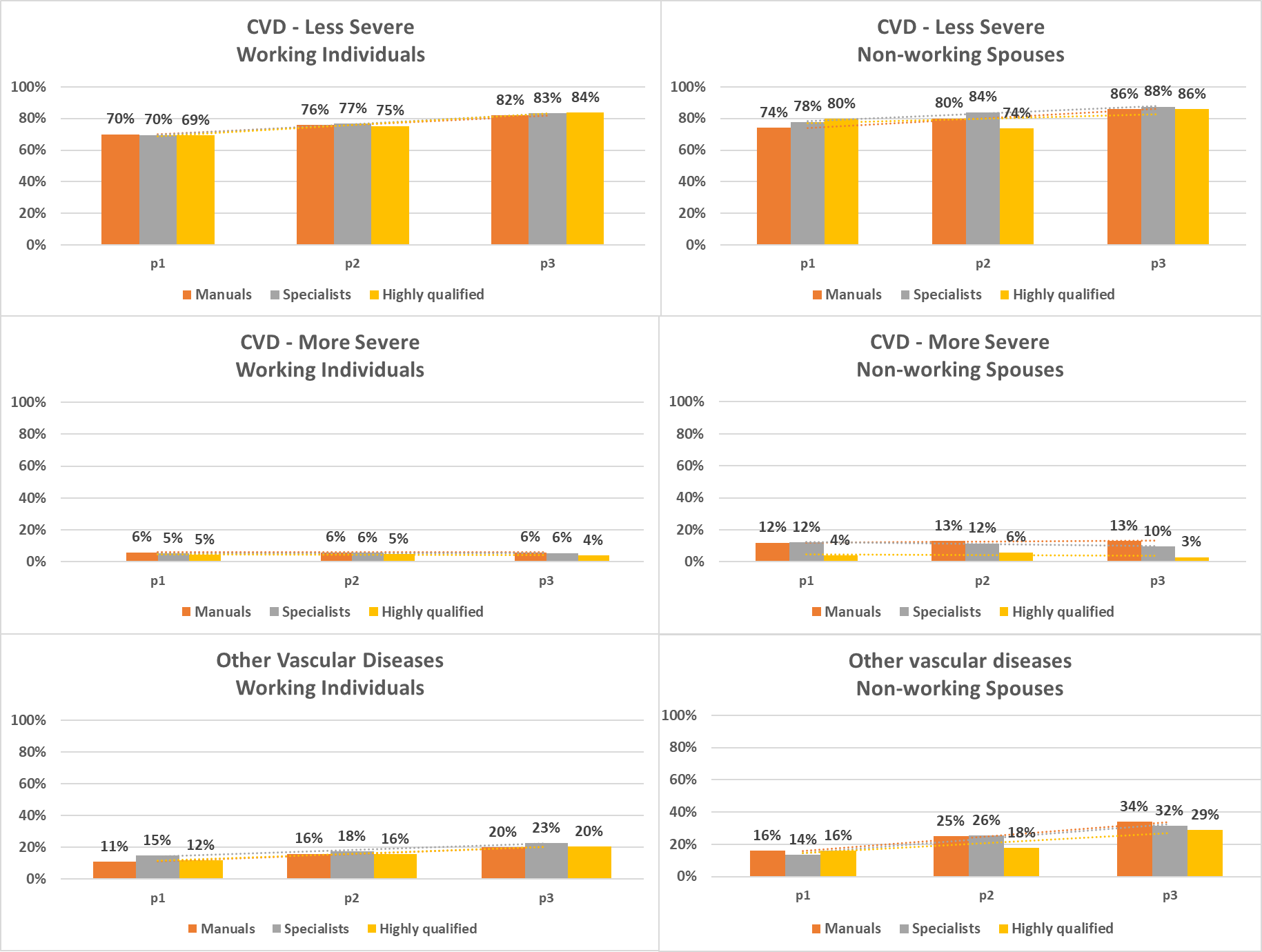
**

**Figure S6.** Predicted probabilities of comorbidity groups over the three time periods for **women**, stratified by **occupation** and population subgroup. ***Time periods*** p1: 2005-2007, p2: 2010-2012, p3:2015-2017. ***Comorbidities*** Less severe CVD: Hypertension, Hyperlipidemia, Cardiac insufficiency; More severe CVD: Myocardial infarction, Stroke, Angina Pectoris; Other vascular diseases: Nephropathy, Neuropathy, Retinopathy.

**
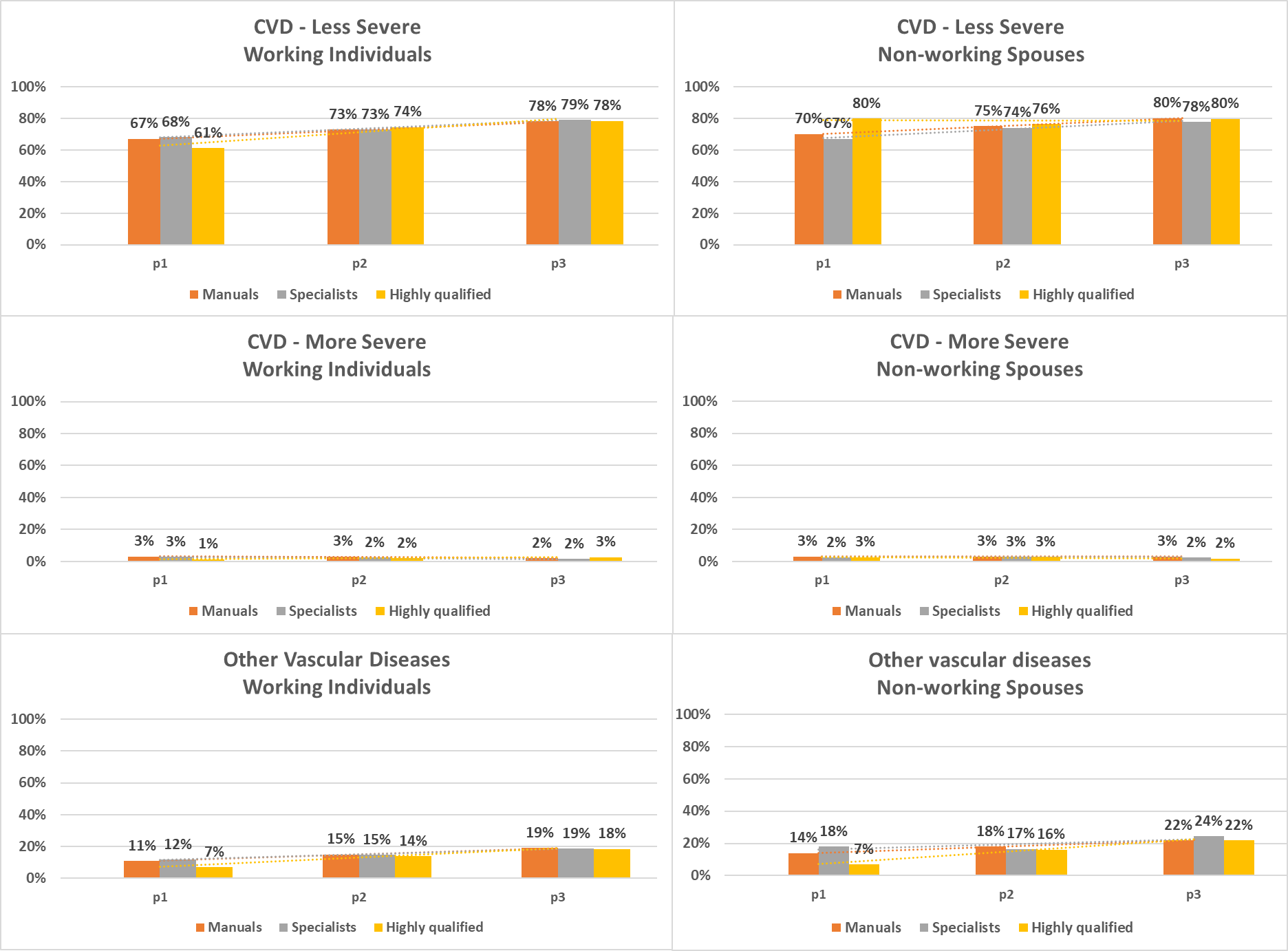
**

**Figure S7.** Predicted probabilities of the number of comorbidities over the three time periods for **men**, stratified by **occupation** and population subgroup. ***Time periods*** p1: 2005-2007, p2: 2010-2012, p3:2015-2017.


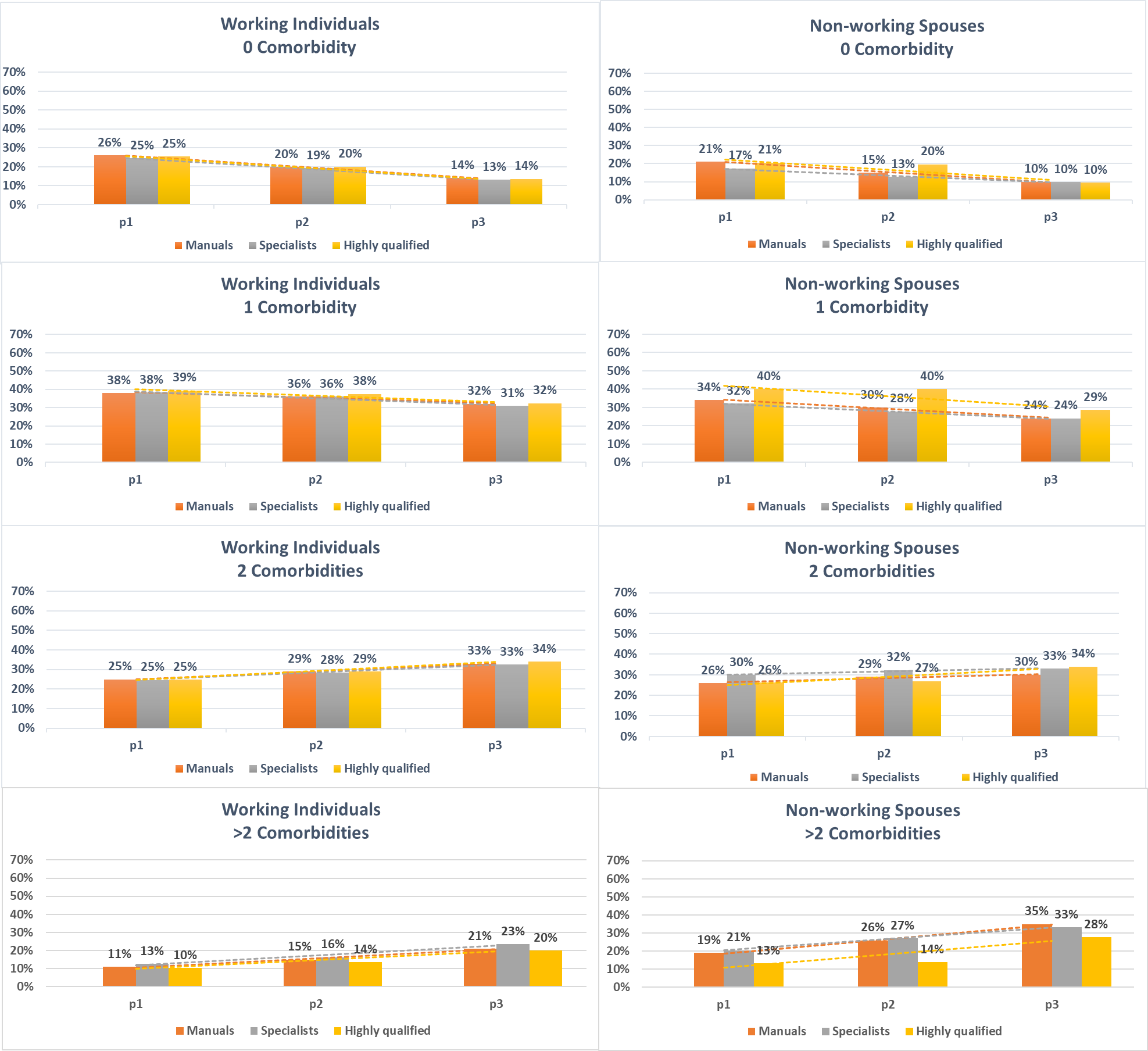


**Figure S8.** Predicted probabilities of the number of comorbidities over the three time periods for **women**, stratified by **occupation** and population subgroup. ***Time periods*** p1: 2005-2007, p2: 2010-2012, p3:2015-2017.


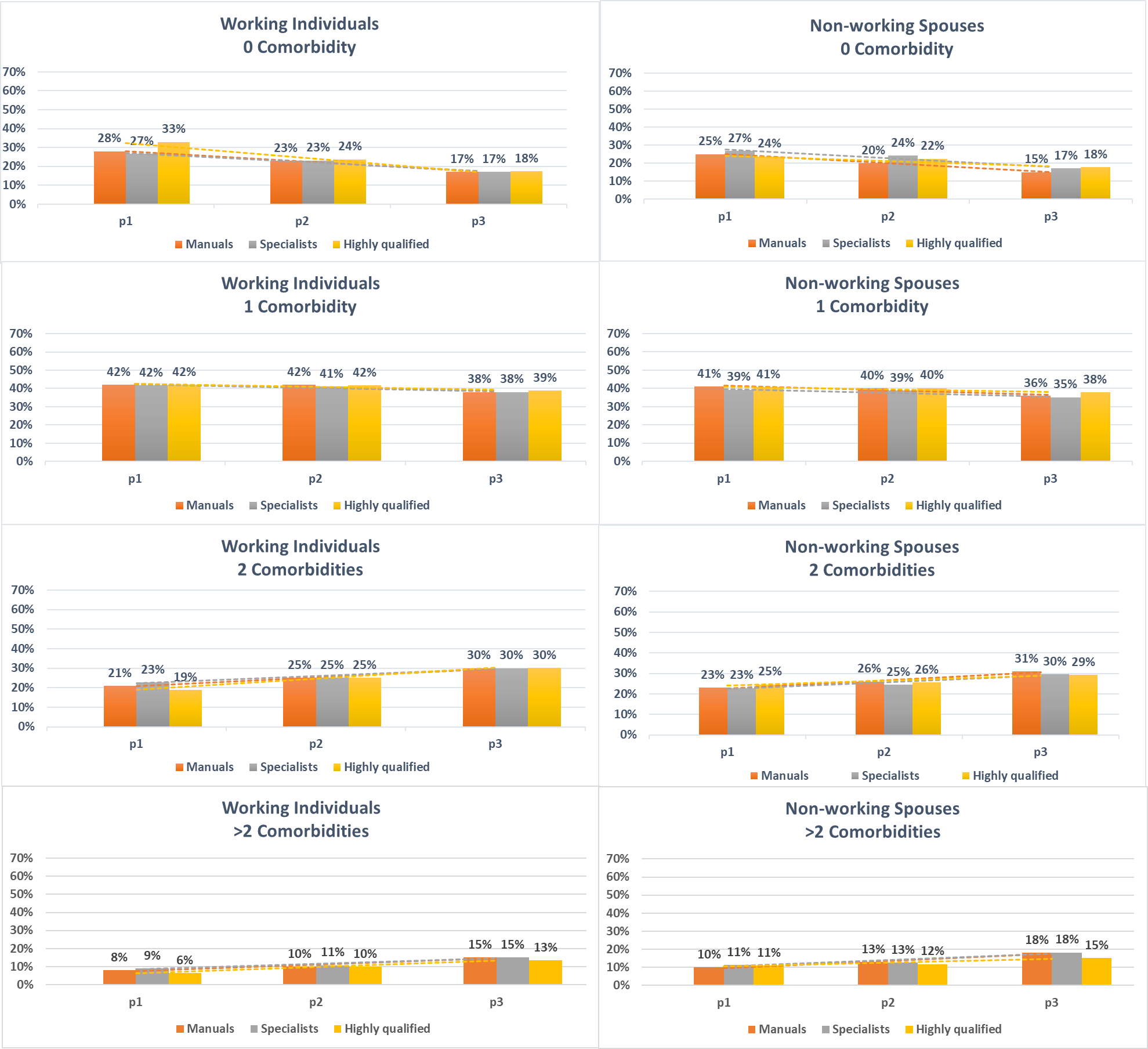


**References**

1. Safieddine B, Sperlich S, Epping J, Lange K, Geyer S. Development of comorbidities in type 2 diabetes between 2005 and 2017 using German claims data. Scientific reports. 2021;11(1):1-10.

2. Bundesministerium der Justiz. Sozialgesetzbuch (SGB) Sechstes Buch (VI) - Gesetzliche Rentenversicherung - (Artikel 1 des Gesetzes v. 18. Dezember 1989, BGBl. I S. 2261, 1990 I S. 1337) 2023 [Available from: https://www.gesetze-im-internet.de/sgb_6/.

3. Bundesministerium für Arbeit und Soziales. Verordnung über maßgebende Rechengrößen der Sozialversicherung für 2017 [Available from: https://www.bmas.de/SharedDocs/Downloads/DE/Meldungen/2016/referentenentwurf-zur-sozialversicherungs-rechengroessenverordnung.pdf?__blob=publicationFile&v=2.
